# Hydrostatic Pressure Helps to Cultivate an Original Anaerobic Bacterium From the Atlantis Massif Subseafloor (IODP Expedition 357): *Petrocella atlantisensis* gen. nov. sp. nov.

**DOI:** 10.3389/fmicb.2019.01497

**Published:** 2019-07-16

**Authors:** Marianne Quéméneur, Gaël Erauso, Eléonore Frouin, Emna Zeghal, Céline Vandecasteele, Bernard Ollivier, Christian Tamburini, Marc Garel, Bénédicte Ménez, Anne Postec

**Affiliations:** ^1^Aix-Marseille Université, Université de Toulon, CNRS, IRD, MIO UM110, Marseille, France; ^2^INRA, US 1426, GeT-PlaGe, Genotoul, Castanet-Tolosan, France; ^3^Université de Paris, Institut de Physique du Globe de Paris, CNRS UMR 7154, Paris, France

**Keywords:** Atlantis Massif, subseafloor, oceanic crust, serpentinization, anaerobic culture, hydrostatic pressure

## Abstract

Rock-hosted subseafloor habitats are very challenging for life, and current knowledge about microorganisms inhabiting such lithic environments is still limited. This study explored the cultivable microbial diversity in anaerobic enrichment cultures from cores recovered during the International Ocean Discovery Program (IODP) Expedition 357 from the Atlantis Massif (Mid-Atlantic Ridge, 30°N). 16S rRNA gene survey of enrichment cultures grown at 10–25°C and pH 8.5 showed that *Firmicutes* and *Proteobacteria* were generally dominant. However, cultivable microbial diversity significantly differed depending on incubation at atmospheric pressure (0.1 MPa), or hydrostatic pressures (HP) mimicking the *in situ* pressure conditions (8.2 or 14.0 MPa). An original, strictly anaerobic bacterium designated 70B-A^T^ was isolated from core M0070C-3R1 (1150 meter below sea level; 3.5 m below seafloor) only from cultures performed at 14.0 MPa. This strain named *Petrocella atlantisensis* is a novel species of a new genus within the newly described family *Vallitaleaceae* (order *Clostridiales*, phylum *Firmicutes*). It is a mesophilic, moderately halotolerant and piezophilic chemoorganotroph, able to grow by fermentation of carbohydrates and proteinaceous compounds. Its 3.5 Mb genome contains numerous genes for ABC transporters of sugars and amino acids, and pathways for fermentation of mono- and di-saccharides and amino acids were identified. Genes encoding multimeric [FeFe] hydrogenases and a Rnf complex form the basis to explain hydrogen and energy production in strain 70B-A^T^. This study outlines the importance of using hydrostatic pressure in culture experiments for isolation and characterization of autochthonous piezophilic microorganisms from subseafloor rocks.

## Introduction

The subseafloor biosphere remains largely unexplored, although estimated as a huge reservoir for prokaryotic life (Whitman et al., 1998; Orcutt et al., 2011; Kallmeyer et al., 2012). Exploration of the rock-hosted subseafloor biosphere is especially very challenging and carried out through costly ocean drilling programs, which still must face several technical difficulties, such as poor core recovery and microbial contamination. In addition, the very low microbial cell density, around 10^4^ cells per gram of rock at North Pond (Jørgensen and Zhao, 2016) or even less in the Atlantis Massif (Früh-Green et al., 2018) (respectively 22 and 30°N along the Mid-Atlantic Ridge), the usually low growth rate, and the lack of knowledge on the physiology and metabolism of the prokaryotes living in these extreme environments, hamper the attempt at cultivating them. As a result, the subseafloor prokaryotic cultivability was estimated below 0.1% of total microscopically counted cells (D’Hondt et al., 2004). Moreover, the hydrostatic pressure is an important physical parameter of these deep-sea environments but was often neglected in previous subseafloor cultivation studies. To date, most of the enrichment tests performed on rocks recovered during ocean drilling programs were made at atmospheric pressure and failed to obtain microbial growth after first incubations or subcultures (Santelli et al., 2010; Hirayama et al., 2015). Both hydrostatic and lithostatic pressures in deep-sea and deep subseafloor environments (increasing by about 10 and 30 MPa km^-1^, respectively (Schrenk et al., 2010)) have an impact on microbial growth, metabolism and physiology, thus on the cultivability of microorganisms (Bartlett et al., 2007; Lauro and Bartlett, 2007; Parkes et al., 2009; Takai, 2011; Picard and Daniel, 2013). Moreover, the importance of high HP for deep-sea microorganisms cultivation is now well established (Tamburini et al., 2013). In this work, HP incubation systems were used to cultivate and study physiology of microorganisms inhabiting the underexplored oceanic crustal biosphere.

**FIGURE 1:**
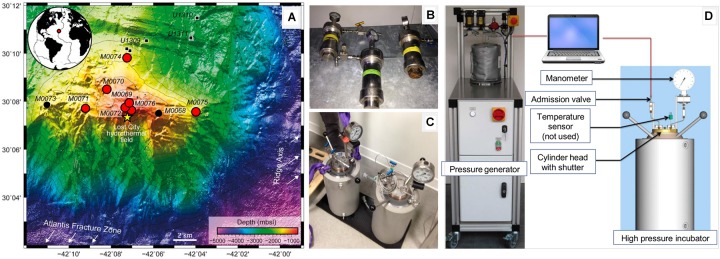
Bathymetric map of the Atlantis Massif southern wall showing the location of the sites drilled during the IODP Expedition 357 [**A**; modified from (Rouméjon et al., 2018)] and the high-pressure devices used in this study **(B–D)**. On the map, the red circles indicate the sites considered in this study, the small black circles correspond to other holes drilled during the expedition, the small black squares show the Holes drilled during IODP Expeditions 304-305 (Site U1309) and the yellow star indicates the Lost City Hydrothermal Field. On the right, the photographs show high-pressure vessels (0.2 l in **B** or 5 l in **C**) used for storage and incubation, respectively. High-pressure vessels were connected to a piloted pressure generator **(D)** allowing linear increase for pressurization (or decrease for depressurization) of the hydrostatic pressure (0.5 MPa sec^-1^) by the programmable computer-driven system [see details in (Tamburini et al., 2009)].

The Atlantis Massif, a prominent underwater oceanic core complex of nearly 4 000 m high, hosts the famous Lost City Hydrothermal Field (LCHF) (Kelley et al., 2005). It is composed of deep crustal (gabbro) and upper mantle rocks (peridotite) that have been exposed at the ocean floor as a result of tectonic plates drifting and large active faulting (Früh-Green et al., 2018). Serpentinization of ultramafic mantle rocks by deeply circulating seawater produces heat and generates alkaline fluids enriched in hydrogen (H_2_), methane (CH_4_), short-chain alkanes and small organic acids, representing possible carbon and energy sources to fuel life in the absence of light and contributing to global biogeochemical cycles (Levin et al., 2016). Such environmental conditions and ecosystems may have prevailed on early Earth or other planets (Martin et al., 2008). In this context, the main objective of the IODP Expedition 357 “Atlantis Massif Serpentinization and Life” was to explore the extent and activity of the subseafloor biosphere in a young ultramafic substratum (Früh-Green et al., 2016, Früh-Green et al., 2017d). During this expedition, series of boreholes were drilled across the Atlantis Massif at different water depths and distances of LCHF (Figure 1), in order to evaluate how subseafloor microbial communities may vary depending on the age of the lithosphere, its alteration and the hydrothermal activity.

The emblematic LCHF located on top of the Atlantis Massif, at 800 meters below sea level (mbsl), exhibits high carbonate chimneys discharging alkaline hydrothermal fluids at moderate temperature (∼ pH 11 and 90°C) and high levels of dissolved H_2_ (1-15 mM) and CH_4_ (1-2 mM) as the most obvious manifestation of underground serpentinization reactions (Schrenk et al., 2013). The unique microbial communities living inside the porous chimney structures are dominated by a single *Methanosarcinales* phylotype (*Archaea*), with *Proteobacteria* and *Firmicutes* (*Bacteria*) (Schrenk et al., 2013). This peculiar ecosystem is assumed to picture an “open window on deep serpentinizing hydrothermal system” (Lang et al., 2018). However, due to the lack of core samples from the LCHF basement, direct experimental evidences supporting this assumption are missing. Only one study on the microbiology of the Atlantis Massif (IODP Expeditions 304-305, Hole U1309D) reports that the gabbroic layers host a low diversity of proteobacterial lineages (*Alpha-, Beta-* and *Gammaproteobacteria*) hypothetically degrading hydrocarbons and fixing carbon and nitrogen with the potential for anaerobic respiration of nitrate, sulfate and metals (Mason et al., 2010). To date no cultivated microorganism has been reported from these ecosystems to attest the occurrence of these metabolisms, and most of the deep-subsurface microorganisms detected so far were refractory to cultivation.

The primary goal of this study was to explore the diversity of cultivable prokaryotic communities of the Atlantis Massif subseafloor from a unique collection of rock cores composed in various proportions of carbonate, basalt, serpentinized peridotite and gabbro (Figure 1 and Supplementary Figure 1). To increase our chance of success, we used a high-pressure incubation system (Figure 1) to mimic *in situ* HP at the sampling sites and the various anaerobic metabolisms likely to be present were targeted based on literature surveys (Mason et al., 2010; Schrenk et al., 2013). We used next-generation sequencing (NGS) of 16S rRNA gene amplicons to explore the cultivable microbial diversity of Atlantis Massif core samples incubated at atmospheric or *in situ* HP. We report novel anaerobes isolated from rock-hosted subseafloor ecosystems and describe the phenotypic and genomic features of strain 70B-A^T^, the first isolate from the Atlantis Massif subseafloor obtained from high-pressure cultures. Finally, we propose it to represent a novel species of a new bacterial genus within the newly described family *Vallitaleaceae* (order *Clostridiales*, phylum *Firmicutes*).

## Materials and Methods

### Rock Sample Collection

A unique set of rock samples was recovered during IODP Expedition 357 “Atlantis Massif Serpentinization and Life,” which took place from October to December 2015 onboard the *RRS James Cook* (Früh-Green et al., 2016). Nine core samples were investigated in this study and were collected using two seabed rock drills (i.e., the British Geological Survey RockDrill2 (RD2) and the Meeresboden-Bohrgerät 70 (Mebo) from the Center for Marine Environmental Sciences, MARUM, University of Bremen, Germany). Drilling sites were located at varying distances away from LCHF and the ridge axis: one site drilled on the eastern part of the southern wall of the Atlantis Massif (Site M0075), three sites located in the central section <1 km north of Lost City (Sites M0069, M0072, and M0076), two sites located north toward the central dome of the massif (Sites M0070 and M0074), and one site on the western end (Site M0071) (Table 1 and Figure 1). Drilled sites were located at a water depth ranging from 820 to 1 568 mbsl (Table 1). Sites M0070 and M0074 targeted the mafic, plutonic domain drilled during IODP Expeditions 304-305 at Site U1309 while the other sites targeted the serpentinite basement of the Atlantis Massif. Lithologies associated with each core sample are described in Table 1 (photographs are shown in Supplementary Figure 1). During drilling, potential contamination was quantified using perfluorocarbon tracer and sampling was achieved under strict contamination controls onboard and offshore (Orcutt et al., 2017). Only samples with no detectable contamination were used for this study (Früh-Green et al., 2017c). Primary procedures, as well as core handling and processing used during the offshore and onshore phases of IODP Expedition 357 are detailed in (Früh-Green et al., 2017c). Details on studied sites according to their location on the Atlantis Massif are provided in (Früh-Green et al., 2017a,b,e,f).

**Table 1:** IODP expedition 357 samples used in this study (see photographs in Supplementary Figure 1).

Site	Hole	Core	Section	Interval (cm)		Depth (mbsf)		Lithology	Depth (mbsl)	Latitude, longitude
				Top	Bottom	Top	Bottom			
**69**	A	4	1	25	35	5.41	5.51	Foraminiferal carbonate sand	850	30.13240, –42.12003
**69**	**A**	**9**	**2**	**111**	**118.5**	**14.48**	**14.55**	**Serpentinized dunite with carbonate veins**	**850**	**30.13240,**–**42.12003**
**70**	**C**	**3**	**1**	**71**	**79**	**3.41**	**3.49**	**Carbonate-hosted basalt breccia**	**1,140.5**	**30.14240,**–**42.13657**
**71**	**C**	**5**	**CC**	**2**	**5**	**7.81**	**7.84**	**Serpentinized harzburgite**	**1,400**	**30.12860,**–**42.15312**
**72**	**B**	**7**	**1**	**106**	**119**	**10.05**	**10.18**	**Metagabbro**	**820**	**30.12990,**–**42.12205**
										
**74**	**A**	**1**	**1**	**50**	**60**	**0.50**	**0.60**	**Carbonate sand (highly disturbed during core recovery)**	**1,550**	**30.16442,**–**42.12192**
**75**	**B**	**3**	**1**	**54**	**74**	**4.52**	**4.72**	**Rubble: Talc schist, minor serpentinite**	**1,568**	**30.12750,**–**42.06617**
**76**	B	3	1	90	102	4.34	4.46	Metadolerite	760	30.12702, –42.11775


*CC stands for core catcher, mbsf stands for meters below seafloor, mbsl stands for meters below sea level. Samples for which enrichment cultures succeeded are highlighted in bold.*

### Processing and Storage of Core Samples Used in This Study

**FIGURE 2:**
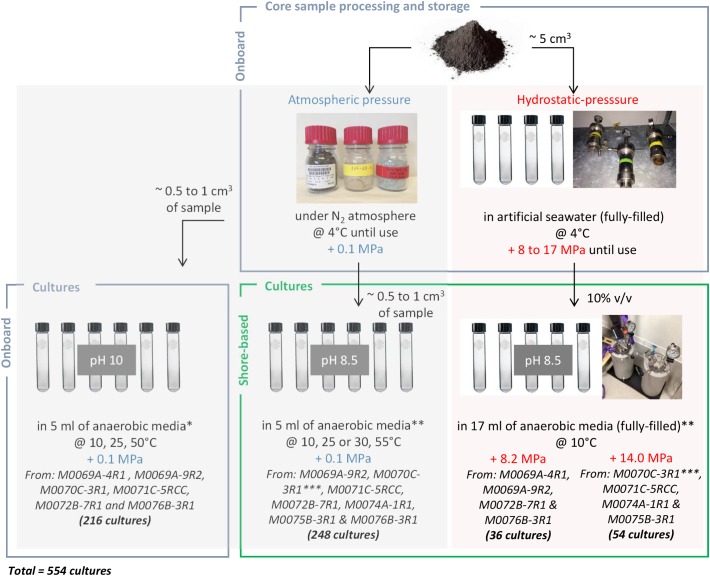
Schematic view of the sampling processing and the cultures initiated at 0.1, 8.2 and 14.0 MPa, onboard and onshore. A total of 554 cultures was performed, in duplicate (except if too small rock quantity); controls were uninoculated tubes. ^∗^Fermentation (F); acetotrophic (MAcH2), hydrogenotrophic (MH2), and methylotrophic (MMet) methanogenesis; acetotrophic (SAc) and hydrogenotrophic (SH2) sulfate reduction. ^∗∗^Fermentation (F1), oligotrophy (F2), sulfate reduction from acetate or lactate (SRB1) and formate or H_2_/CO_2_ (SRB2), methanogenesis by acetoclasty or methylotrophy (MET1) and hydrogenotrophy (MET2), and iron reduction (FE) (Supplementary Table 2). ^∗∗∗^Black basaltic and white carbonated parts dissociated.

Onboard, the processing of the core sample was done at 10°C. The exterior of the core sample was flamed to eliminate potential contaminants, then sliced and smashed into small pieces using a flamed chisel. These subsamples were crushed using a sterile stainless steel mortar and pestle, under sterile and anaerobic conditions. Approximately 5 cm^3^ of the powdered rock sample were transferred into Hungate screw tubes, fully filled (∼17 ml) with sterile artificial seawater (40 g l^-1^ solution of Sea Salts, Sigma Aldrich) and stored in high pressure vessels (Top Industrie SA, France) at *in situ* HP conditions (from 8 to 17 MPa, depending on the water depths of the sites) by means of manual hydraulic pump and stored at 4°C until processing in the shore-based laboratory (Figure 2). Extra powder samples were transferred in 100 ml Schott bottles under a N_2_ gas atmosphere, then stored at 4°C and atmospheric pressure (0.1 MPa) until processing in the onboard and shore-based laboratories (Figure 2). Details on HP devices are provided in (Tamburini, 2006; Tamburini et al., 2009, 2013).

### Culture Media Preparation and Enrichment Culture Conditions

The composition of the culture media is detailed in the Supplementary Table 1. After dissolution of its components, the culture medium was boiled under a stream of O_2_-free N_2_ gas, cooled to room temperature, dispensed into Hungate tubes, flushed under N_2_/CO_2_ (80/20, v/v) and then sterilized by autoclaving. The media were completed by addition of sterile stock solutions of Na_2_CO3 8% (w/v), MgCl_2_6H_2_O 50 g l^-1^, Balch oligo elements solution, and Na_2_S9H_2_O 3% (w/v) injected through the tube septum (volumes are indicated in Supplementary Table 1).

Onboard, culture media were inoculated in duplicates in Hungate tubes at 0.1 MPa headspace of N_2_/CO_2_ or H_2_/CO_2_ (80/20, v/v) using approximately 0.5–1 cm^3^ of rock as inoculum in 5 ml of anaerobic media (with the following abbreviations: F, for fermenters; MAcH2, MH2, MMet, for acetotrophic, hydrogenotrophic and methylotrophic methanogens; SAc and SH2, for acetotrophic and hydrogenotrophic sulfate reducers; Supplementary Table 1). Culture media pH were adjusted to 10 with 1M KOH as detailed in (Früh-Green et al., 2017c) and incubations were done at three temperatures: 10°C (the average temperature of sea bottom waters), 25 and 50°C (as we hypothesized a temperature gradient between hot hydrothermal fluid and cold subseafloor) A total of 216 tubes were inoculated with core samples M0069A-4R1, M0069A-9R2, M0070C-3R1, M0071C-5RCC, M0072B-7R1 and M0076B-3R1 (6 samples × 2 replicates × 3 temperatures × 6 media) (Figure 2).

In the shore-based laboratory (MIO), series of culture media (composition in Supplementary Table 1) were used to target the following metabolisms: fermentation (F1), oligotrophy (F2), sulfate reduction from acetate or lactate (SRB1) and formate or H_2_/CO_2_ (SRB2), methanogenesis by acetoclasty or methylotrophy (MET1) and hydrogenotrophy (MET2), and iron reduction (FE). The ISO 10390:2005 protocol, dedicated to measure soil pH, was used here for rock samples: powdered rocks were suspended in KCl 1M (1/5, v/v), and pH of the suspension was measured. The mean pH was 8.5 (below a pH of 10, which was the first assumption for onboard enrichment cultures) thus the pH of the shore-based culture media was accordingly adjusted to pH 8.5. Hungate tubes for incubation at 0.1 MPa contained 5 ml of medium, thus about 12 ml of gas headspace (N_2_/CO_2_ or H_2_/CO_2_, 80/20, v/v), while those used in HP incubation were filled with 17 ml of medium (no headspace) (Khelaifia et al., 2011). Cultures were run in duplicates, except for samples M0069A-4R1 and M0074A-1R1 due to the low amount of available material. 248 enrichment cultures at 0.1 MPa were incubated at 10°C, 25°C (or 30°C) and 55°C from core samples M0069A-9R2, M0070C-3R1 (black basaltic and white carbonated parts dissociated), M0071C-5RCC, M0072B-7R1, M0074A-1R1, M0075B-3R1, and M0076B-3R1 (8 samples × 2 replicates × 3 temperatures × 6 media -all except SRB2-, MET2 without replicate and only at 30°C). 36 enrichment cultures were launched at 8.2 MPa and 10°C from core samples M0069A-4R1, M0069A-9R2, M0072B-7R1 and M0076B-3R1 (the last two in duplicate) using the 6 onshore media (all except F2), while 54 enrichment cultures were launched at 14.0 MPa and 10°C from core samples M0070C-3R1 (black basaltic and white carbonated parts dissociated), M0071C-5RCC, M0074A-1R1 and M0075B-3R1 using the 6 onshore media (all except F2).

All cultures carried out under HP were incubated at 10°C (corresponding to the average temperature of sea bottom waters) (Figure 2). Shortly, culture tubes were placed for incubations in two HP 5 l inox incubators which were totally filled with distilled water, the septum transmitting the hydrostatic pressure inside the culture, before pressure was increased. A piloted pressure generator (see details in (Tamburini et al., 2009)) was used for accurate pressurization and depressurization (ramping rate = 3 MPa/min) (Figure 1D).

For each culture condition, a tube of medium not inoculated was incubated in parallel as negative control, to verify the absence of microbial contamination. The experimental procedure is summarized in Figure 2.

After 1 week incubation onboard and 1 month incubation (and more) in shore-lab experiments, the enrichment cultures were checked for growth by observation of cells stained with SYBR^®^ Green I (Molecular Probes) 1X using an epifluorescence microscope Nikon ECLIPSE E600. The headspace gas, in the cultures that have one, were analyzed by gas chromatography (Shimadzu GC 8A instrument) to evaluate H_2_ production or consumption and CH_4_ production, as described in (Mei et al., 2014).

### Strain Isolation and Analyses

Positive cultures were serially diluted at 1/10 of the volume up to 10^-9^ dilution in Hungate screw tubes containing 4.5 ml of anaerobic growth media plus agar (1.6% final) maintained liquid at 55°C in a water-bath. Inoculated tubes were then quickly cooled down while spinning using a tube spinner according to the roll-tube technique (Hungate, 1969). Colonies were picked up in an anaerobic gloves-box and used as an inoculum for the next series of dilution. This procedure was repeated three times before the strain culture was deemed pure. Morphological features of the isolates were examined using a microscope Nikon ECLIPSE E600 under phase contrast condition. Procedure for analyzing cell structures, the G+C content of genomic DNA, as well as cellular fatty acids and polar lipids composition, are given in Supplementary Methods.

### Determination of Optimal Growth Conditions of Strain 70B-A^T^

Experiments to determine growth ranges of strain 70B-A^T^ were performed in duplicates or in triplicates at 0.1 MPa using Hungate tubes containing 5 ml of F1 medium (for pH, temperature and salinity) or at 14.0 MPa using BM medium (i.e., mineral base of the F1 medium) supplemented with 0.2 g l^-1^ yeast extract and 20 mM glucose as sole carbon source. Potential substrates were tested in duplicates by addition into the BM medium (containing 0.5 g l^-1^ yeast extract). Details on experimental procedures are given in Supplementary Methods. Growth was determined by measuring optical density (OD) at 600 nm (Cary 50 UV-Vis spectrophotometer; Varian). End-products of metabolism were measured by high-performance liquid chromatography (HPLC) and gas phase chromatography after 2-weeks of incubation at 30°C (instruments details in (Mei et al., 2014)). Graphical and statistical analyzes of growth curves (OD *versus* time) of strain 70B-A^T^ were performed with XLSTAT 2018.6 (Microsoft Excel add-in program). After checking data normality, the parametric Student’s *t*-test or non-parametric Mann-Whitney test were respectively used to estimate if the differences observed in maximum OD values (OD_max_) or maximal growth rates between the cultures performed at 0.1 and 14.0 MPa were significant.

### DNA Extraction and 16S rRNA Gene Sequencing

Fast DNA^®^ kit for soil and FastDNA^®^ kit were used respectively to extract DNA from enrichment cultures and isolated strain, according to the manufacturer recommendations (MP Biomedical). DNA from enrichment cultures was used for PCR amplification targeting the V3-V4 region of the 16S rRNA gene using the primers Pro341F and Pro805R (Takahashi et al., 2014); amplicons were sequenced by Illumina MiSeq at MrDNA (TX, United States). 16S rRNA genes from isolated strains were amplified using the primers 27F and 1492R (Lane, 1991) and the PCR products were sent to GATC Biotech AG (Germany) for Sanger sequencing.

### Whole Genome Sequencing of Strain 70B-A^T^

For sequencing the genome of strain 70B-A^T^, high molecular weight DNA was extracted from a 500 mL culture cell pellet by using a phenol-chloroform-isoamyl alcohol-based method as previously described (Marteinsson et al., 1995). Genomic sequencing was performed at the GenoToul platform (Toulouse, France) by combining long reads technology of Oxford Nanopore to ease assembling (GridION) and high coverage provided by short paired-end reads obtained with Illumina (MiSeq) technology. The methodological details on sequencing, read processing and *de novo* assembly of genome are provided in Supplementary Methods. The genome was annotated using the MicroScope plateform (Vallenet et al., 2017).

### Bacterial Community Composition and Phylogenetic Analysis

Raw reads were first merged with PEAR v0.9.6 and trimmed when the quality scores were less than 20 for two consecutive bases. Only sequences with a length greater than 35 bp were retained for further analyses. Chimera sequences were identified and removed using vsearch (v2.3.4). The clustering to Operational Taxonomic Units (OTUs) (>97%) and their quality filtration were carried out using QIIME 1.9.1 (Caporaso et al., 2010) as described in (Frouin et al., 2018). Taxonomic assignment of the filtered OTUs was performed using the Uclust method against the SILVA database release for QIIME (v.123). Heatmap.3 package in R was used to build the heatmap (R Core Team, 2017).

Phylogenetic analyses and trees based on 16S rRNA gene sequences were conducted in MEGA7 (Kumar et al., 2016) using MUSCLE program (Edgar, 2004) for multiple alignment, as described by (Postec et al., 2015).

### Data Deposition

MiSeq V3-V4 amplicons raw data were submitted to the Sequence Read Archive under SRA Study SRP148750, as part of the BioProject PRJNA448822. The GenBank/EMBL/DDBJ accession number for the 16S rRNA gene sequence (1490 bp) of strain 70B-A^T^ is KY969626. The genome sequence was deposited to the European Nucleotide Archive under study accession number PRJEB28585^1^, and Genbank accession number LR130778. The type strain 70B-A^T^ was deposited in DSMZ and JCM culture collections under accession numbers DSM_105309^T^ = JCM 32078^T^ respectively.

## Results

### Summary of Enrichment Cultures

A total of 554 anaerobic enrichment cultures were performed from IODP 357 core samples and monitored for about 1 year (Figure 2). Onboard, the first positive enrichment cultures were obtained after 2-week incubation from M0069A-9R2 using MAcH2 media at 25°C, 0.1 MPa and pH 10 (in the range of low temperature serpentinizing fluids pH values). No microbial growth was observed in other alkaliphilic cultures initiated onboard after 1-year regular monitoring, whatever the temperature and medium conditions tested. Back to the home lab, the first enrichment cultures at 0.1 MPa were obtained after 1-month incubation from M0070C-3R1 at 25°C in F1 and FE media, but only scarce cells were detected by epifluorescence microscopy from M0069A-9R2, M0071C-5RCC, M0072B-7R1 and M0074A-1R1 cultures after 2 months under similar conditions. Dividing cells were observed at 10°C in the same media than at 25°C but after longer incubation (≥2 months). No microbial growth was detected in any of the enrichment cultures at 55°C, neither from M0069A-4R1 (carbonate sand) or M0076B-3R1 (serpentinite) samples, in any conditions. At 14.0 MPa, active cells were observed from M0070C-3R1 sample after 1 month with all the tested media (more cells with F1 one), while scarce active cells were observed with M0075B-3R1 in F1 and SRB1 media. At 8.2 MPa, a slight growth was detected after 1-year incubation from M0072B-7R1 (using F1 and SRB1 media).

### Phyla Diversity in Enrichment Cultures

Microbial diversity was analyzed by MiSeq 16S rRNA gene sequencing for a selection of 51 enrichment cultures, listed and described in Supplementary Table 2. A total of 462,162 sequences clustered in 415 OTUs was obtained.

**FIGURE 3:**
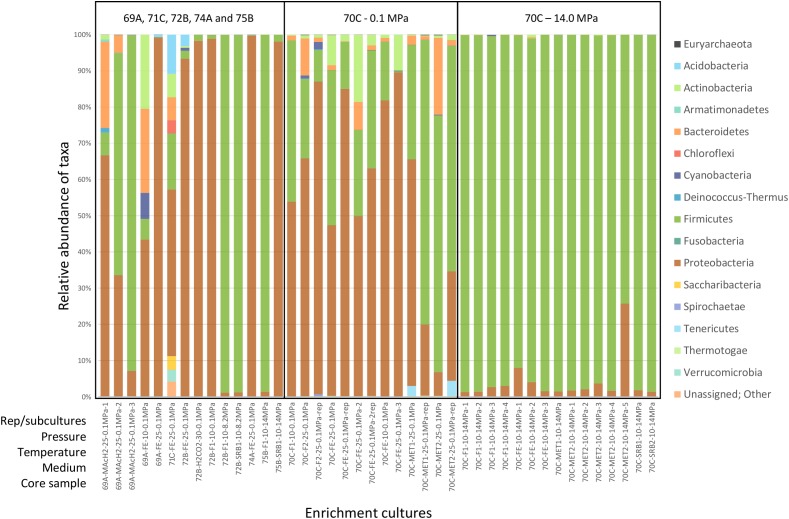
Phylum-level microbial diversity in enrichment cultures from Atlantis Massif core samples. Analysis based on 16S rRNA gene sequences (V3–V4 region). 69A, 70C, 71C, 72B, 74A and 75B respectively stand for M0069A-9R2, M0070C-3R1, M0071C-5RCC, M0072B-7R1, M0074A-1R1 and M0075B-3R1 core samples. The sample names used in this study and their description in SRA (study SRP148750) are shown in Supplementary Table 2. Rep stands for replicate. Pressure is expressed in MPa, temperature in °C. F, SRB, FE, MET & MAcH2 correspond to culture media targeting fermenters, sulfate reducers, iron reducers and methanogens, respectively (Supplementary Table 1).

*Firmicutes* and *Proteobacteria* were largely dominant in most conditions tested (Figure 3). *Actinobacteria*, *Bacteroidetes*, *Cyanobacteria* and *Deinococcus-Thermus* were also abundant in M0069A-9R2 cultures (MAcH2 and FE media, 10 and 25°C, 0.1 MPa) while *Acidobacteria*, *Chloroflexi*, *Saccharibacteria* and *Verrucomicrobia* were abundant in a M0071C-5RCC culture (FE medium, 25°C, 0.1 MPa). Enrichment cultures from M0072B-7R1, M0074A-1R1 and M0075B-3R1 contained only *Firmicutes* and *Proteobacteria*, except for sample M0072B-7R1 in FE medium at 25°C and 0.1 MPa culture containing also *Acidobacteria*, *Actinobacteria* and *Cyanobacteria*. In M0070C-3R1 cultures at 14.0 MPa, *Firmicutes* accounted for 74% to 99% of the microbial community (with the second most abundant phylum being *Proteobacteria*), while a more diverse community exhibiting *Bacteroidetes*, *Actinobacteria*, *Tennericutes* and *Cyanobacteria* members was detected at 0.1 MPa.

*Archaea* represented less than 0.2% of the total reads and were not detected in all cultures. *Methanosarcinales* affiliated to ANaerobic MEthane oxidizing *Archaea* (ANME-3) were found in M0069A-9R2, M0070C-3R1 and M0074A-1R1 [and already recognized in Lost City Hydrothermal chimneys (Brazelton et al., 2006)], together with rare OTUs assigned to the hyperthermophilic sulfur-reducing genus *Thermococcus* and the thermoacidophilic genus *Thermoplasma* (Table 2).

**Table 2:** Relevant potential metabolisms/phenotypes of operational taxonomic units (OTUs) retrieved from anaerobic enrichment cultures from Atlantis Massif core samples (based on 16S rRNA gene analysis).

Potential metabolisms/phenotypes	OTUs affiliation (Genera)	Hole occurrences	Total reads abundance (%)
Hydrocarbon degraders	***Acinetobacter***, ***Halomonas***, *Marinobacter*, *Alcanivorax*, *Alicyclobacillus*, ***Sphingomonas***	69A, 70C, 71C, 72B, 74A, 75B	7.02
Iron reducers	***Shewanella***	69A, 70C, 72B	5.37
Thermophiles (hydrothermal origin)	*Caminicella*, *Desulfotomaculum*, *Desulfovibrio*, *Gaeilla*, *Mesoaciditoga*, *Thermicanus*, *Thermoanaerobacterium*, *Thermococcus^∗^*, *Thermoleophilia*, *Thermoplasma^∗^*, *Thermus*, *Thiomicrospira*, *Truepera*, *Vallitalea*	69A, 70C, 71C, 72B, 74A, 75B	1.44
Alkaliphiles	*Acetoanaerobium*, *Alkalibacterium*, *Alkaliphilus, Desulfonatronum*, *Thioalkalispira*	69A, 70C, 71C, 72B, 74A, 75B	0.55
Sulfate reducers	*Desulfacinum*, *Desulfobacter*, *Desulfovibrio*, *Desulfonauticus*, *Desulfonatronum*, *Desulfotomaculum*	69A, 70C, 71C, 72B, 74A	0.23
*S*-compounds oxidation	*Arcobacter*, *Sulfurimonas*, *Sulfurovum*, *Acidithiobacillus*, *Sulfitobacter*, *Thioalkalispira*, *Thiobacillus*, *Thiomicrospira*	69A, 70C, 71C, 72B, 74A, 75B	0.22
H_2_ consumers	*Hydrogenophaga*, *Thiobacillus*, *Desulfotomaculum*	69A, 70C, 71C, 72B, 74A	0.06
Methyl or CH_4_ consumers	Unknown genus *Methylococcales* (Marine Methylotrophic Group 1), *Methanosarcinales* (ANME-3)^∗^	69A, 70C, 71C, 72B, 74A, 75B	0.03
Iron oxidizers	*Mariprofundus*, *Marinobacter*, *Thiobacillus*	69A, 70C, 71C, 72B, 74A, 75B	0.01


*Dominant OTUs (>10% total reads) are indicated in bold and archaeal taxa using an asterisk. 69A, 70C, 71C, 72B, 74A, and 75B respectively stand for M0069A-9R2, M0070C-3R1, M0071C-5RCC, M0072B-7R1, M0074A-1R1, and M0075B-3R1 core samples.*

### Dominant Cultivated Microorganisms: Abundant OTUs

**FIGURE 4:**
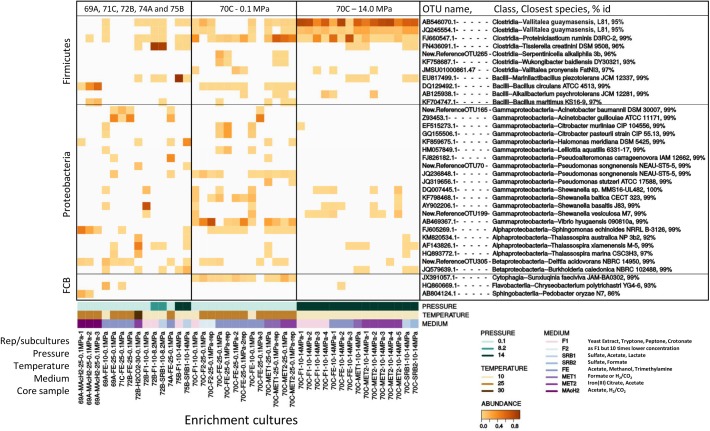
Heatmap showing the distribution of dominant OTUs (>10% total reads) in anaerobic enrichment cultures from Atlantis Massif subseafloor rocks. OTU name, its class affiliation, name of its closest species and 16S rRNA gene percentage identity (% id) with this closest homolog are indicated on the right. 69A, 70C, 71C, 72B, 74A and 75B respectively stand for M0069A-9R2, M0070C-3R1, M0071C-5RCC, M0072B-7R1, M0074A-1R1 and M0075B-3R1 core samples. Rep stands for replicate. Pressure is expressed in MPa, temperature in °C. F, SRB, FE, MET & MAcH2 correspond to culture media targeting fermenters, sulfate reducers, iron reducers and methanogens, respectively (Supplementary Table 1). FCB stands for *Fibrobacteres*/*Bacteroidetes/Chlorobi* group.

Only the most abundant OTUs (>10% of total reads), representing the most likely cultivated members, are presented in Figure 4. Enrichment cultures from M0069A-9R2 were dominated at 25°C and 0.1 MPa by *Acinetobacter* (*Gammaproteobacteria*), *Bacillus* (*Bacilli*), *Sphingomonas* (*Alphaproteobacteria*) and *Sphingobacteriia (Bacteroidetes)*. Interestingly, an effect of the HP was observed for M0072B-7R1 cultures since *Tissierella* (*Clostridia*) largely dominated at 8.2 MPa while *Thalassospira* (*Alphaproteobacteria*; using H_2_/CO_2_), *Shewanella* and *Acinetobacter* (*Gammaproteobacteria*) were dominant at 0.1 MPa. Besides, *Marinilactibacillus* and *Halomonas*, also found in other deep ecosystems and including some piezophilic representatives (Kaye et al., 2004; Toffin et al., 2005), were cultivated at 14.0 MPa from M0075B-3R1 (Figure 4).

Most of the successful enrichment cultures were obtained from carbonate-hosted basalt breccia samples of M0070C-3R1 core (1150 mbsl, north of the Atlantis Massif). Marked differences in the microbial diversity of the cultures were observed depending on the incubation pressure. At 0.1 MPa, the dominant genera in M0070C-3R1 cultures were *Proteiniclasticum* (*Clostridia*), *Vibrio*, *Shewanella*, *Acinetobacter*, *Citrobacter* and *Pseudomonas* (belonging to *Gammaproteobacteria*) (Figure 4). *Sunxiuqinia* (*Cytophagia*) and *Tessaracoccus* (*Actinobacteria*; not shown, <10%), less abundant in the cultures, have been previously reported in deep biosphere environments, as well as *Shewanella* (Toffin et al., 2004; Finster et al., 2009; Picard and Daniel, 2013; Takai et al., 2013). In contrast, cultures from M0070C-3R1 incubated at 14.0 MPa were dominated mainly by 2 OTUs of an unknown genus, related to *Vallitalea* genus (*Clostridia*), representing up to 98% of the reads in a culture (Figure 4). *Proteiniclasticum, Alkalibacterium* (*Firmicutes*) with *Shewanella* were also found as dominant OTUs in M0070C-3R1 cultures at 14.0 MPa (also found at 0.1 MPa).

Most of the identified microorganisms seem to develop on heterotrophy in our enrichment cultures. Additionally, potential iron reducers (e.g., *Shewanella*) were identified as dominant, while potential H_2_-consumers (*Hydrogenophaga, Thiobacillus*) and/or sulfate-reducers (*Desulfotomaculum*) were only observed as rare OTUs (Table 2).

### Strain Isolation

In this study, a total of fourteen strains were isolated and affiliated to *Gammaproteobacteria*, *Actinobacteria*, *Bacillales* and *Clostridiales* (Supplementary Figure 2).

Three very similar isolates (100% 16S rRNA gene identity) affiliated to *Tissierella* genus (97% 16S rRNA gene identity with *T. creatinophila, Clostridiales*) were obtained from M0072B-7R1 cultures at 8.2 MPa using media SRB1 and F1 after 1-year incubation. At 25°C and 0.1 MPa, *Sphingobacteriia* initially enriched from M0069A-9R2 (in MAcH_2_ medium) and accounting for 48% of the microbial community, was outcompeted by *Bacillus* after only two subcultures. A strain affiliated to *Bacillus circulans* (99% 16S rRNA gene identity) was finally isolated with LB-Tris medium from M0069A-9R2 (Supplementary Figure 2B).

The rest of the strains (10/14) were isolated from M0070C-3R1 subcultures. Among them, the strain 70-CrotoS2-10-2 isolated at 25°C and 0.1 MPa using F1 medium was closely affiliated to *Tessaracoccus oleiagri* and *T. profundii* (99% 16S rRNA gene identity). A positive enrichment culture at 25°C and 0.1 MPa with FE medium was also used as an inoculum for serial dilution on roll-tubes from which dark brown colonies, likely indicating iron reduction, formed after 4 days up to the dilution 10^-7^. Four of these colonies were assigned to *Citrobacter freundii* (99% 16S rRNA gene identity) in the *Enterobacteriaceae* family (class *Gammaproteobacteria*). One of them, strain 70O-Fer25-B, is a motile coccobacillus shown in Supplementary Figure 2A.

Within *Firmicutes*, two strains closely affiliated to *Proteiniclasticum ruminis* (99% identity; type strain from yak rumen) were isolated at 10°C and 0.1 MPa in F1 medium. Finally, six isolates very closely related (sharing >99% 16S rRNA gene identity), were obtained from M0070C-3R1 cultures incubated at 14.0 MPa and 10°C in MET1, MET2 and F1 media. First colonies were obtained in roll-tubes after about 1 month incubation at 10°C and further purified by two additional series end-point dilution in roll-tubes before deemed pure. They were related to *Vallitalea* genus at <93% 16S rRNA gene identity and therefore represente new cultivated members of *Clostridiales* without close described species. One of them, called strain 70B-A^T^, was chosen for complete characterization.

### Characterization of Strain 70B-A^T^

**FIGURE 5:**
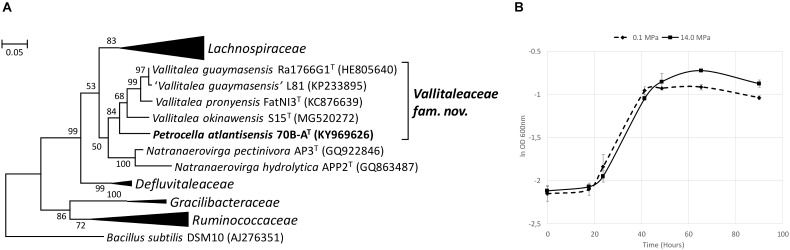
Phylogenetic and physiological characteristics of strain 70B-A^T^. **(A)** Maximum Likelihood phylogenetic tree based on 1203 aligned base pairs of 16S rRNA gene sequences showing the position of strain 70B-A^T^ in the *Clostridiales* order. The Kimura 2-parameter was used, and the analysis involved 34 sequences. *Bacillus subtilis* (AJ276351) was used as an outgroup. Bootstrap values higher than 50% (based on 1 000 replicates) are shown at branch nodes. Accession numbers of type species are indicated in brackets. Bar, 0.05 substitutions per nucleotide. **(B)** Growth curves of strain 70B-A^T^ at 0.1 and 14.0 MPa. Apart of hydrostatic pressure, all other culture conditions were identical (in Hungate tube with no headspace). The BM medium was supplemented with 0.2 g l^-1^ yeast extract and 20 mM glucose. The ln OD _600nm_ average from triplicates is shown here. Error bars are the associated standard deviation.

The 16S rRNA gene sequence of strain 70B-A^T^ was most closely related to members of *Vallitalea* and *Natranaerovirga* genera, with *V. guaymasensis* (Lakhal et al., 2013) and *N. pectinivora* (Sorokin et al., 2012) sharing respectively 92.6 and 90.2% 16S rRNA gene sequence identity with strain 70B-A^T^ (Figure 5A). Strain 70B-A^T^ genomic DNA contained 37.7 mol % of G+C.

The major cellular fatty acids of strain 70B-A^T^ were C16:1 w7c (35.6%), C16:0 (22.1%) and C16:1 w7c DMA (14.3%) (Supplementary Table 3). The main polar lipids consisted of nine phospholipids, one glycolipid, one phosphatidylglycerol and one phosphoglycolipid (Table 3).

**Table 3:** Differential phenotypic and genotypic characteristics between strain 70B-A^T^ and type strains of recognized *Vallitalea* species: *V. guaymasensis^T^* (Kumar et al., 2016), *Natranaerovirga pectinivora^T^* (Schrenk et al., 2010).

Characteristics	70B-A^T^	*V. guaymasensis* Ra1766G1^T^	*N. pectinivora* AP3^T^
Origin	Atlantis Massif subseafloor	Sediment of Guaymas Basin	Soda soils
Temperature growth range (optimum) (°C)	10–35 (25)	20–40 (30–35)	≤43
pH growth range (optimum)	5.6–9.2 (7.8)	6.0–8.0 (6.5–7.5)	8.0–10.5 (9.5–9.7)
Salinity growth range (optimum) (%)	0–8 (2.5)	0.5–7.5 (2–3)	1.2–14.6 (2.0)
Gram	+	-	+
Motility	+	-	-
Spore	-	-	+
Main carbon sources	Carbohydrates	Carbohydrates	Galacturonic acid and pectin
Arabinose	-	+	-
Cellobiose	+	+	-
Fructose	+	-	-
Galactose	+	+	-
Glucose	+	+	-
Glycerol	+	-	-
Lactose	+	-	-
Maltose	+	+	-
Mannitol	+	+	-
Mannose	+	+	-
Pyruvate	+	+	-
Raffinose	+	+	-
Rhamnose	+	-	-
Sucrose	+	+	-
Xylose	-	+	-
Yeast extract	+	+	-
Polar lipids	PL, GL, PG, PGL	PG, DPG, PL, GL	PG, DPG, PL, GL, APL
Dominant fatty acids	C16:1 w7c, C16:0, C16:1 w7c DMA	Anteiso-C15:0, iso-C15:0, anteiso-C15:0 DMA, iso-C15:0 DMA	16:0, 16:1w7c, 18:1w7c
Main fermentation products	Acetate, H_2_, CO_2_, and formate	Acetate, H_2_, and CO_2_	Acetate and formate
**Genomic features**			
Size (base pair)	3,518,882	6,419,149	3,061,098
GC content	37.3%	31.2%	31.3%
Contig nb	1	7	29
Protein-coding genes (CDS)^∗^	3,423	5,600	2,859
CDS with predicted function	2,904	4,043	2,251
rRNA genes (16S, 23S, 5S)	15	17	12
tRNA	56	56	50


*Genomic features of* V. guaymasensis *originated from strain L81 (no data available for the type strain) (Rouméjon et al., 2018). PL, phospholipids; GL, glycolipids; PG, phosphatidylglycerol; PGL, phosphoglycolipid; APL, aminophospholipids; DMA, dimethylacetals; CDS, coding DNA sequence. ^∗^Using MicroScope annotation platform and IMG-ER annotation platform for strain 70B-A^*T*^ and* V. guaymasensis *L81, respectively. Genomes accession numbers:* P. atlantisensis *70B-A^*T*^: PRJEB28585;* V. guaymasensis *L81: NZ_QMDO00000000.1; and *N. pectinovora* AP3: IMG submission ID 191455.*

Strain 70B-A^T^ cells rod shaped (0.4–0.7 μm in diameter and 2.0–12.0 μm in length) during the exponential growth phase (Supplementary Figure 3A) where it is motile through the action of observed polar and lateral monotrich flagella (data not shown). No spore formation was observed. Under unfavorable conditions (e.g., pH 11), morphology changed and showed inflated and deformed cells (Supplementary Figure 3B). The cell wall structure observed by transmission electron microscopy revealed a Gram-positive structure with the presence of a multilayered cell wall without an outer membrane as confirmed by Gram-staining (Supplementary Figure 3C).

Strain 70B-A^T^ grows under strict anaerobic conditions from 0 to 8% (w/v) NaCl (optimum at 2.5% (w/v) of NaCl; Supplementary Figure 4B), 10 to 35°C (optimum at 25°C; Supplementary Figure 4A), and pH 5.6 to 9.2 (optimum at pH 7.4-8.0). HP did not significantly improve its maximum specific growth rate: μ_max_ was in average 0.048 h^-1^ at 0.1 MPa and 0.045 h^-1^ at 14.0 MPa (Figure 5B). However, the maximum OD was significantly higher at 14.0 MPa (0.476 ± 0.016) than at 0.1 MPa (0.422 ± 0.011; Student *t*-test, *p* = 0.003).

Strain 70B-A^T^ was able to use cellobiose, fructose, galactose, glucose, glycerol, lactose, maltose, mannitol, mannose, pyruvate, raffinose, rhamnose, ribose, sucrose, trehalose, tryptone, yeast extract (YE), but not acetate, arabinose, butyrate, casaminoacids, citrate, crotonate, ethanol, formate, lactate, methanol, pectine, peptone, propionate, succinate, TMA, xylose. No growth was observed with only H_2_/CO_2_ or H_2_/CO_2_ and acetate as sole carbon sources. Strain 70B-A^T^ is strictly chemoorganotrophic as confirmed by HPLC analysis of substrate utilization. Growth on carbohydrates resulted in acetate as the main metabolic product, along with formate, H_2_ and CO_2_ (Table 3). Strain 70B-A^T^ was not able to use thiosulfate, sulfate, sulfite, nitrite, nitrate, Fe(III) citrate and elemental sulfur as electron acceptors. Under optimal conditions, the maximal growth rate (on BM medium plus glucose) was 0.048 h^-1^.

Strain 70B-A^T^ has a lower growth temperature range than the type species of *Vallitalea* and *Natranaerovirga* (Table 3). While its pH and salinity ranges as well as its use of carbon source (mainly carbohydrates) are similar to that of *V. guaymasensis*, strain 70B-A^T^ differs by its capacity to utilize fructose, glycerol, lactose and rhamnose (Table 3). Fatty acids profile resembles more that of *N. pectinovora* with abundant C16 and the absence of C15. Unlike the two other strains, 70B-A^T^ displays cell motility and a singular profile of polar lipids and a higher DNA G + C content (Table 3).

### Genome Analysis of Strain 70B-A^T^

**FIGURE 6:**
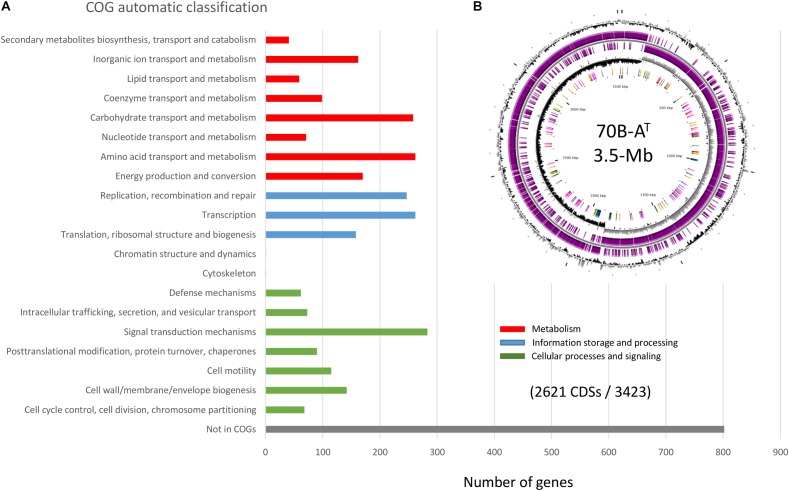
Strain 70B-A^T^ genome analysis. **(A)** Functional categorization of 2621 Coding DNA Sequences (CDS) (out of 3423) based on the Clusters of Orthologous Groups (COGs) database (Galperin et al., 2015), **(B)** Circular genome view.

The complete genome (one single contig) of strain 70B-A^T^ consists of a circular chromosome of 3 518 882 bp, with 37.3% GC content (Table 3). The chromosome contains 3 536 predicted genes, of which 3 423 are protein-coding. The size of the strain 70B-A^T^ genome is considerably smaller than that of *V.* *guaymasensis* L81, 6.42 Mb, and *V. okinawensis*, 5.86 Mb, but in the range of those of the *Natranaerovirga* genomes, 3.06 Mb for *N. pectinivora* and 2.98 Mb for *N. hydrolytica* (Schouw et al., 2018; Sun et al., 2018). Of the 3 423 protein-coding genes, 2 904 were given a predicted function (Figure 6). Carbohydrates and amino acids transport and metabolisms were among the most represented functional categories with respectively 6.3 and 6.1% of the function-predicted genes. In agreement with the culture-based conclusions on the metabolism, the genome of strain 70B-A^T^ encodes several glycosyl hydrolases (β-galactosidase, β-glucosidase, and α-amylase), proteases and peptidases. It contains numerous ABC-transporters of multi-sugars (pentose, hexose, and di-saccharides) and several sugars (mannose, fructose, lactose) phosphotransferase systems (PTS). A complete glycolysis (Embden-Meyerhof) and gluconeogenesis pathways were identified, as well as uncomplete Entner-Doudoroff and pentose phosphate pathways as observed in some Gram positive bacteria and archaea (Conway, 1992). The genes for the complete biosynthesis pathways of all the amino acids are present, except for the L-lysine and L-methionine which are incomplete and thus maybe not operational. Nevertheless, the genome encodes two oligo-peptides and, at least 12 amino-acids ABC transporters with all kind of specificity for polar amino-acids, branched-amino acids, lysine or arginine, and proline-betaine, suggesting these served as substrates for growth. The degradation pathways identified for L-alanine, L-cysteine, L-tryptophan, L-serine and L-threonine lead to pyruvate while those of L-aspartate, L-asparagine, L-Glutamate, and L-Glutamine generate oxaloacetate, fumarate or acetyl-CoA which could aliment an uncomplete citric acid (TCA) cycle (the succinyl-CoA synthase gene is lacking). Pyruvate could also be directly fermented in lactate (by a lactate dehydrogenase), acetate or ethanol (by an alcohol dehydrogenase) or via the common mixed acid fermentation pathway. A putative pyruvate: ferredoxin oxidoreductase catalyzes the formation of acetyl-CoA from the pyruvate, coupled to the reduction of ferredoxin (Fd). Thus, most of the reducing power of the diverse fermentations is transferred to NADH and reduced Fd electron carriers, and we postulate, as recently proposed for *V. guaymasensis* (Schouw et al., 2018), this fuels a hydrogenase to generate H_2_. Indeed, genes for a tetrameric [Fe-Fe] hydrogenase are found as an operon (PATL70BA_1019 to PATL70BA_1022) homologous to *hndA, hndB, hndC, hndD*, encoding a cytosolic NADP-reducing hydrogenase in *Desulfovibrio fructosivorans* (Dermoun et al., 2002). In this sulfate-reducing bacterium, this enzyme could either catalyzes H_2_ oxidation during growth on H_2_ as a sole energy source or produces H_2_ when growing by fermentation of fructose in the absence of sulfate as a terminal electron acceptor. We assume that this tetrameric hydrogenase utilizes the mechanism of flavin-based electron bifurcation (FBEB) (Schuchmann et al., 2018): the exergonic electron flow from reduced Fd to H^+^ drives endergonic electron flow from NADH to H^+^. The archetype of such so-called bifurcative/confurcative hydrogenase was first described in *Thermotoga maritima* (Schut and Adams, 2009). Two additional hydrogenases-encoding genes are present, PATL70BA_1241 is homologous to a 4Fe-4S dicluster domain-containing, poorly characterized, periplasmic hydrogenase found in many anaerobic *Firmicutes*, and PATL70BA_2847, another homolog of the *hndA* subunit gene. Besides, the genes encoding a [FeFe] hydrogenase maturase (HydEFG), involved in the synthesis and incorporation of the di-iron center, were identified. The genome of strain 70B-A^T^ also contains an operon (*rnfC, rnfD, rnfG, rsxE, rsxA, rnfB*) encoding an Rnf complex coupling the translocation of proton/sodium ion across the membrane with the reversible oxidation of reduced ferredoxin with NAD^+^ (Westphal et al., 2018). The Rnf complex could, therefore, serve to generate a proton/sodium gradient while equilibrating the pool of reductants (NADH) produced during sugars or amino-acids fermentation (Buckel and Thauer, 2018; Westphal et al., 2018).

The genome encodes both a V-type ATPase and an F-type of ATPase. The V-type ATPases use ATP to translocate protons across membranes, while F-type ATPases generate ATP *via* proton translocation (Perzov et al., 2001). The genome also contains a cluster of 9 genes (PATL70BA_2417 to 2426) encoding a nitrogenase (*nifH, nifD, nifK, nifE, nifB, nifV*) conferring this bacterium the ability to fix N_2_, which may represent a selective advantage to survive in its challenging habitat. Several clusters of metal or metalloids (Fe, Zn, Cr, Ni, Ar, Hg) transporters genes were also found which could be involved in either import (iron siderophores) or export (detoxication) processes. Finally, the genome contains numerous transposases and integrases genes, of which many belong to putative prophages and two large conjugative integrated plasmids, also called integrons (Mazel, 2006), known as essential agents of bacterial genome evolution.

## Discussion

Today, our knowledge of subseafloor microbial ecosystems is mainly based on data from culture-independent methods such as 16S rRNA barcoding or metagenomics. Although these approaches have been proved incomparable to explore and describe the extent of microbial diversity and model the functioning of these ecosystems, they present only a partial view of the real picture since most of the microorganisms inhabiting these environments are uncultivated (Jungbluth et al., 2016). Subseafloor microorganisms have been mostly recalcitrant to cultivation in laboratory, making their physiological characterization complicated. Most of these microorganisms were isolated from sedimentary rock cores and included mostly aerobes or facultative anaerobes but a few strict anaerobes even though, passed the first tens centimeters depth below the seafloor, these environments are essentially anoxic (Toffin and Alain, 2014). Previous attempts at cultivating anaerobes from various subseafloor sediments often led to the isolation of the same few ‘generalist’ bacteria belonging to genera counting numerous species and strains encompassing a broad spectrum of environmental conditions (Ciobanu et al., 2014; Parkes et al., 2014).

In this study, our main goal was to cultivate anaerobic microorganisms inhabiting the rocky subseafloor associated with active serpentinization characterized by circulation of high pH fluids enriched in H_2_, CH_4_ and other organic molecules abiotically produced. To the best of our knowledge, the only cultivated anaerobes were isolated by our team from a shallow submarine serpentinite-hosted hydrothermal system, analogous to the LCHF, located in the Prony bay (New Caledonia) (Ben Aissa et al., 2014, 2015; Mei et al., 2014, 2016; Bes et al., 2015). In the present study, putative moderately halo-alkaliphilic bacteria (related to *Alkalibacterium*, *Halomonas*, *Tissierella* and the proposed genus *Petrocella*) were enriched and in some case isolated in cultures carried out at pH 8.5, while the only isolate obtained from the cultures at pH 10 (in the common range of pH values observed in low temperature serpentinizing systems) was a strain of *Bacillus circulans*, a facultative anaerobe, spore-forming, frequently detected in soils, but no obligate alkaliphile as targeted by the pH conditions. These results may be explained by the relatively low pH values (7.82–7.87) measured in the hole bottom waters, with maximum values of pH 7.98 recorded in Hole M0070C (Früh-Green et al., 2018). Anaerobic H_2_-consumers, sulfate-reducers or methanogens (main metabolisms detected at LCHF) were not abundant in cultures despite the use of various culture conditions targeting these metabolisms and the elevated H_2_ and CH_4_ levels measured in bottom waters (5-73 and 2-5 nM, respectively, at Site M0070 where gas bubbles were observed issuing from the hole, see Figure 13 in Früh-Green et al. (2018)).

In such rock-hosted subseafloor ecosystems, cell abundance is well known to be exceptionally low making contamination a crucial issue, and cultivation of indigenous microorganism very challenging. Cell abundance in the IODP 357 core samples was very low ranging from tens to thousands of cells cm^-3^ with a maximum in Hole M0069A sediments (up to 1.6 × 10^4^ cells cm^-3^), before decreasing rapidly to <10^2^ cells cm^-3^ in the underlying basement rocks (Früh-Green et al., 2018). In this study, growth was observed after a relatively short incubation time (1 month) in M0069A-9R2 and M0070C-3R1 enrichment cultures at 0.1 MPa and 25°C which may be due to contamination as suggested by the affiliation (at 99% identity) of the dominant OTU to genus or species corresponding to ubiquitous bacteria (*Citrobacter freundii, Proteiniclasticum ruminis, Bacillus circulans*; Figure 4 and Supplementary Figures 2A,B) found in a wide range of habitats not specific of the deep biosphere or serpentinizing systems. In contrast with cultures at 0.1 MPa, utilization of HP induced a drastic change in the structure of the bacterial communities for all combination of samples and cultures conditions (medium and incubation temperature), even at modest HP of 8.2 MPa, when compared to their counterpart incubated at atmospheric pressure (0.1 MPa gas headspace) as shown in Figure 3. Such phenomena has been since confirmed in our lab (Garel et al., 2019). Thus, one may assume that even moderately elevated HP inhibits growth of surface waters microorganisms and other contaminants that in these conditions are outcompeted by true deep sea/rock inhabitants. HP allowed the cultivation of novel microorganisms such as a potential new *Tissierella* species obtained at 8.2 MPa from M0072B-7R1 core (metagabbro) and a new *Clostridiales* lineage obtained at 14.0 MPa with M0070C-3R1 core sample (carbonate-cemented basaltic breccia) (Figure 4 and Supplementary Figures 2A,B). Moreover, the isolate 70B-A^T^, representing this new lineage, exhibits a strict anaerobic growth at 14.0 MPa making it very unlikely to originate from seawater or drilling equipment, but instead it may come from anoxic subseafloor. Strain 70B-A^T^, as well as its closest neighbors belonging to the *Vallitalea* genus, were adapted to live in the deep-sea and subseafloor environments, both impacted by various kinds of hydrothermal activity in different geographical and geodynamic area (Arctic Mid-Ocean Ridge, Northeast and Southwest Pacific Ocean, East China Sea and now Mid-Atlantic Ridge). Here, strain 70B-A^T^ displayed maximum cell density (OD_max_) under HP conditions (at 14 MPa). Knowing that the maximum population density (OD_max_) can strongly influence the definition of the optimal growth conditions (Martini et al., 2013), strain 70B-A^T^ can therefore be considered as a piezophile even if further studies are needed to characterize its lifestyle under HP.

Mason et al. (2010) gave the first insight into the microbial community inhabiting the Atlantis Massif subseafloor using molecular approaches. They identified bacteria usually related to hydrocarbon-rich environment and recognized hydrocarbon degraders (Mason et al., 2010). Here, potential hydrocarbon degraders (*Halomonas*, *Sphingomonas*) were also detected as dominant in our anaerobic enrichments. The hydrocarbons detected in rocks of the Atlantis Massif may have a marine origin but might also originate from abiotic organic synthesis associated with serpentinization (Delacour et al., 2008; Hickok et al., 2018; Ménez et al., 2018), which may potentially sustain the endolithic microbial community. Interestingly, the closest neighbors of strain 70B-A^T^ belonging to *Vallitalea* genus originated from (i) the serpentinizing hydrothermal system of Prony (PHF; New Caledonia) (Ben Aissa et al., 2014), (ii) the Guaymas basin, a deep hydrothermally influenced ecosystem rich in hydrocarbons (Lakhal et al., 2013) and (iii) Loki’s Castle, a hydrocarbon-rich hydrothermal field (Schouw et al., 2016). However, the role of these microorganisms in hydrocarbon degradation (Schouw et al., 2016) has not yet been established (Schouw et al., 2018). Culture experiments showed that strain 70B-A^T^, like strains *V. guaymasensis* Ra1766G1^T^ and L81, and *V. pronyensis* strain FatNI3^T^, does not use alkanes for growth but, instead, can degrade various carbohydrates and proteinaceous compounds, suggesting this is a metabolic feature common to members of the novel *Vallitaleaceae* family. Genome analysis of strain 70B-A^T^ confirmed the utilization of sugars which are fermented mostly in acetate, CO_2_ and H_2_ *via* the glycolysis and suggests that strain 70B-A^T^ is able to import oligo-peptides and amino acids and to convert them to acetate *via* oxidation to pyruvate and acetyl-CoA. The presence of a putative electron bifurcative/confurcative hydrogenase and an ion-motive Rnf complex, enabling ferredoxin-based pathways, seems to be crucial for energy conservation in members of the proposed family *Vallitaleaceae*. Interestingly, the closest homologs of this novel hydrogenase, and Rnf complex were detected in the same metagenome-assembled genomes (MAG) of dominant uncultivated *Firmicutes* from deep terrestrial subsurface sediments (Hernsdorf et al., 2017).

In conclusion, this study shows that hydrostatic pressure helps to enrich novel anaerobes from subseafloor rocks. The new isolate *Petrocella atlantisensis* 70B-A^T^ is considered to represent a novel species of a novel genus within a novel proposed family *Vallitaleaceae*. It is a halotolerant, psychrotolerant, mesophilic and piezophilic chemoheterotroph able to use carbohydrates, proteinaceous compounds and potentially amino acids. Strain 70B-A^T^ represents the first cultivated and characterized representative of the Atlantis Massif and will constitute a model strain to further study microbial adaptation to the deep subseafloor in a context of serpentinizing ultramafic rocks.

### Description of *Vallitaleaceae* fam. nov.

Cells were rod shaped, obligately anaerobic, mesophilic, neutrophilic and marine. Capable of fermentation (no electron acceptor identified), using cellobiose, galactose, glucose, maltose, mannose, raffinose, sucrose and yeast extract, but not acetate (contrarily to both *Natranaerovirga* species). Originating from hydrothermal fields and/or deep oceanic subsurface habitats rich in hydrocarbon or related to serpentinization process. The family contains the type genus *Vallitalea* and the genus *Petrocella*.

### Description of *Petrocella* gen. nov.

*Petrocella* (Pe.tro.cel’la N. L. n. *petra* a rock, stone; L. fem. n. *cella* a cell; N. L. fem. n. *Petrocella* a rod isolated from a rock). Gram-positive, motile, non-sporulating, marine and mesophilic rods. Fermentative and obligate anaerobe, able to use a wide range of sugars. It belongs to the order *Clostridiales* (phylum *Firmicutes*). The DNA G+C content of the type strain of the type species is 37.7 mol%. The type species is *Petrocella atlantisensis.*

### Description of *Petrocella atlantisensis* sp. nov.

*Petrocella atlantisensis* (a.tlan.tis.en’sis. N. L. fem. adj. *atlantisensis* originated from the Atlantis Massif). It displays the following features in addition to those listed for the genus description. Cells are approximately 2–12 μm long and 0.4–0.7 μm wide, occurring singly or in pairs. Cells are motile and possess polar or lateral monotrich flagella. No spore was observed. Growth occurs at 10–35°C (optimum 25°C), at pH 5.6–9.2 (optimum pH 7.4–8.0) and with 0–8% (w/v) NaCl (optimum 2.5%). Yeast extract is required for growth. Cellobiose, fructose, galactose, glucose, glycerol, lactose, maltose, mannitol, mannose, pyruvate, raffinose, rhamnose, ribose, sucrose, trehalose, tryptone and yeast extract are used as electron donors, but not acetate, arabinose, butyrate, casamino acids, citrate, crotonate, ethanol, formate, H_2_/CO_2_, H_2_/CO_2_ + acetate, lactate, methanol, pectine, peptone, propionate, succinate, TMA, xylose. None of the following electron acceptors was used: elemental sulfur, sulfate, thiosulfate, sulfite, nitrate and nitrite. The major fatty acids are C16:1 w7c, C16:0 and C16:1 w7c DMA. The type strain, 70B-A^T^, ( = DSM 105309^T^ = JCM 32078^T^) was isolated from rocks drilled in the Atlantis Massif (Mid-Atlantic Ridge, 30° N).

## Data Availability

The datasets generated for this study can be found in SRA, and ENA or Genbank, SRP148750 (MiSeq V3-V4 amplicons raw data), and PRJEB28585 or LR130778 (whole genome sequence of strain 70B-A^T^).

## Author Contributions

AP wrote the manuscript in collaboration with MQ. MQ, GE, and AP designed the experiments. MQ collected the samples. MQ, AP, EZ, MG, and CT carried out the high-pressure experiments. EF performed the microbial diversity analyses. CV performed the genome sequencing. All authors contributed to the discussion and the writing of the manuscript.

## Conflict of Interest Statement

The authors declare that the research was conducted in the absence of any commercial or financial relationships that could be construed as a potential conflict of interest.

## Footnotes

1 http://www.ebi.ac.uk/ena/data/view/PRJEB28585
