# Supplementary material for: Hydrostatic Pressure Helps to Cultivate an Original Anaerobic Bacterium From the Atlantis Massif Subseafloor (IODP Expedition 357): Petrocella atlantisensis gen. nov. sp. nov
Source: Front Microbiol. 2019 Jul 16;10:1497. doi: 10.3389/fmicb.2019.01497 (PMC6647913; doi:10.3389/fmicb.2019.01497)
Supplement: Supplementary file 1 [file Data_Sheet_1.pdf]

Hydrostatic pressure helps to cultivate an original anaerobic bacterium from the Atlantis Massif subseafloor (IODP Expedition 357): *Petrocella atlantisensis gen. nov.* sp. nov.

Marianne Quéméneur[1], Gaël Erauso[1], Eléonore Frouin[1], Emna Zeghal[1], Céline Vandecasteele[2], Bernard Ollivier[1], Christian Tamburini[1], Marc Garel[1], Bénédicte Ménez[3], Anne Postec[1]*

Supplementary Methods

Analysis of cell structures, G+C content of genomic DNA, cellular fatty acids and polar lipids composition of strain 70B-A^T

Morphological characteristics and purity of isolated strains were examined using an epifluorescence microscope Nikon ECLIPSE E600 under phase contrast conditions. Cell structures of strain 70B-A^T deposited on Cu-grids were observed using a FEI Tecnai G2 transmission electron microscope (TEM) operating at 200 kV. Exponentially grown cells were negatively stained during 15 min with 2% (w/v) sodium phosphotungstate (pH 7.0) without rinsing for observation of flagella. For observation of the cell wall, cells were first fixed during 20 min with 10% (v/v) glutaraldehyde, stained overnight with osmium tetraoxide 2% (w/v) and embedded in EMbed-812 resin (Electron Microscopy Sciences). Embedded specimens were sliced into ultrathin sections (90 nm in thickness) using a Leica EM UC7 ultramicrotome, placed on Cu TEM grid and stained during 3 min with 2% (w/v) uranyl acetate and 1 min with 1% (w/v) lead before observation.

The G+C content of strain 70B-A^T genomic DNA was determined at the Identification Service of DSMZ (Deutsche Sammlung von Mikroorganismen und Zellkulturen, Braunschweig, Germany). DNA was isolated and purified by chromatography on hydroxyapatite (Cashion et al., 1977) and the G+C content was determined by using high performance liquid chromatography (Mesbah et al., 1989).

The determination of cellular fatty acids and polar lipids composition was performed at DSMZ. After extraction using a modified version of the method of Miller (Miller, 1982; Kuykendall et al., 1988), fatty acids were separated by gas chromatography (GC model 6890N, Agilent Technology) using the Microbial Identification system (MIDI, Sherlock^{TM} Version 6.1; database, TSBA40) (Sasser, 1990).

Details on the determination of optimal growth parameters and substrate consumption of strain 70B-A^T

Temperature, pH and salinity growth ranges and optima of strain 70B-A^T were estimated at atmospheric pressure (0.1 MPa) using Hungate tubes containing 5 ml of F1 medium (Supplementary Table 1). Water baths were used to obtain incubation temperatures ranging from

4°C to 70°C. To determine NaCl requirement, a range of 0 to 9% (w/v) NaCl was tested. The pH of the medium (*i.e.* 5.0, 5.6, 6.0, 6.3, 6.8, 7.4, 8.0, 8.6, 9.2 and 9.9) was adjusted with anaerobic sterile stock solutions of 1M HCl (for low pH values), 10% (w/v) $NaHCO_3$ or 8% (w/v) Na_2CO_3 (for high pH values). MES (10 mM) was used as buffering agent for growth tests at pH 5.0, 5.6 and 6.0 and PIPES (10 mM) for growth tests at pH 6.3 and 6.8. Cultures were performed in duplicate, and then used as inoculum for a second series of cultures under the same experimental conditions for growth rates' determination. Growth was determined by measuring the optical density at 600 nm ($OD_{600\ nm}$) using a Cary 50 UV-Vis spectrophotometer (Varian).
Growth at atmospheric (0.1 MPa) and HP (14.0 MPa) pressure was tested in triplicates at 30°C under the same conditions (*i.e.* in 17 ml Hungate tubes without any gas phase and the same pre-culture used as inoculum for both pressures) using the BM medium (*i.e.* mineral base of the F1 medium; Supplementary Table 1) supplemented with 0.2 g l^{-1} yeast extract and 20 mM glucose as sole carbon source.
Different carbon substrates were tested in duplicate by addition into the BM medium (supplemented with 0.5 g l^{-1} yeast extract) of sugars, organic acids and alcohols at 20 mM final concentration and complex proteinaceous matter at 4 g l^{-1}. Electron acceptors (sulfate, thiosulfate, sulfite, nitrate and nitrite) were tested each at 10 mM final concentration, and elemental sulfur at 10 g l^{-1}.

Details on whole genome sequencing of 70B-A^{T} strain
Genomic sequencing was performed at the Genotoul GeT-PlaGe core facility (INRA Toulouse, France). Oxford Nanopore Technology (ONT) library was prepared according to the manufacturer's instructions "1D gDNA selecting for long reads (SQK-LSK108 ONT)". At each step, DNA was quantified using the Qubit™ dsDNA HS Assay Kit (Life Technologies). DNA purity was evaluated using a Thermo Scientific™ NanoDrop™ and size distribution and degradation were assessed using the High Sensitivity DNA Fragment Analysis Kit (AATI). Purification steps were performed using AMPure XP beads (Beckman Coulter). For one flowcell, 7.5 µg of DNA was sheared at 20 kb using the Megaruptor® system (Diagenode). A DNA damage repair step was performed on 5 µg of sample. Then a END-repair and dA tail of double stranded DNA fragments was performed and adapters were ligated to the library. Library (0.15 pmols) was loaded on a R9.5 flowcell (ONT) and sequenced on GridION instrument (ONT) within 48 h.
Illumina paired-end (PE) reads were prepared according to Illumina's protocols using the TruSeq Nano DNA LT Library Prep Kit (Illumina). Briefly, DNA were fragmented by sonication and adaptors were ligated to be sequenced. 8 cycles of PCR were applied to amplify libraries. Library quality was assessed using Fragment Analyzer (AATI) and libraries were quantified by quantitative PCR using the Kapa Library Quantification Kit (Roche). DNA sequencing experiments have been performed on a MiSeq instrument (Illumina) using a PE read length of 2×150 pb with the MiSeq Reagent Kit Micro v2.

Details on reads' processing and *de novo* assembly of strain 70B-A^{T} genome
Fast5s from ONT sequencing were obtained with the MinKNOW ONT software (1.10.24-1 version) and were basecalled with Albacore Sequencing Pipeline Software (from ONT, 2.1.10 version). Reads passing the internal test were used for subsequent analysis. Porechop 0.2.1 (https://github.com/rrwick/Porechop) with the "–discard_middle" option was used for adaptor trimming. Illumina PE reads were processed with trim_galore 0.4.0 (https://github.com/FelixKrueger/TrimGalore) to trim adaptor sequences. Nanopore reads were assembled using Canu 1.7 (Koren et al., 2017) with the "genomeSize=3.5 m" and

“minReadLength=3000” options. For Nanopore-only assembly, one output contig was obtained, then polished twice using Pilon 1.22 (Walker et al., 2014) with the “--mindepth 25” option. The contig was finally circularized using Circlator 1.5. (Hunt et al., 2015).

Supplementary References

Cashion, P., Holder-Franklin, M. A., McCully, J., and Franklin, M. (1977). A rapid method for the base ratio determination of bacterial DNA. *Anal. Biochem.* 81(2)**,** 461-466.

Hunt, M., Silva, N. D., Otto, T. D., Parkhill, J., Keane, J. A., and Harris, S. R. (2015). Circlator: automated circularization of genome assemblies using long sequencing reads. *Genome Biol.* 16(1)**,** 294. doi: 10.1186/s13059-015-0849-0.

Koren, S., Walenz, B. P., Berlin, K., Miller, J. R., Bergman, N. H., and Phillippy, A. M. (2017). Canu: scalable and accurate long-read assembly via adaptive k-mer weighting and repeat separation. *Genome Res.* 27(5)**,** 722-736. doi: 10.1101/gr.215087.116.

Kuykendall, L. D., Roy, M. A., O'Neill, J. J., and Devine, T. E. (1988). Fatty acids, antibiotic resistance, and deoxyribonucleic acid homology groups of *Bradyrhizobium japonicum*. *Int. Syst. Bacteriol.* 38(4)**,** 358-361. doi: 10.1099/00207713-38-4-358.

Mesbah, M., Premachandran, U., and Whitman, W. B. (1989). Precise measurement of the G+C content of deoxyribonucleic acid by high-performance liquid chromatography. *Int. J. Syst. Bacteriol.* 39**,** 159-167. doi: 10.1099/00207713-39-2-159.

Miller, L. T. (1982). Single derivatization method for routine analysis of bacterial whole-cell fatty acid methyl esters, including hydroxy acids. *J. Clin. Microbiol.* 16(3)**,** 584-586.

Sasser, M. (1990). Identification of bacteria by gas chromatography of cellular fatty acids. MIDI Technical Note 101. Microbial ID, Inc., Newark, DE, USA.

Walker, B. J., Abeel, T., Shea, T., Priest, M., Abouelliel, A., Sakthikumar, S., et al. (2014). Pilon: An integrated tool for comprehensive microbial variant detection and genome assembly improvement. *PLoS One* 9(11)**,** e112963. doi: 10.1371/journal.pone.0112963.
