# Supplementary material for: Hydrostatic Pressure Helps to Cultivate an Original Anaerobic Bacterium From the Atlantis Massif Subseafloor (IODP Expedition 357): Petrocella atlantisensis gen. nov. sp. nov
Source: Front Microbiol. 2019 Jul 16;10:1497. doi: 10.3389/fmicb.2019.01497 (PMC6647913; doi:10.3389/fmicb.2019.01497)
Supplement: Supplementary file 2 [file Data_Sheet_2.pdf]

Hydrostatic pressure helps to cultivate an original anaerobic bacterium from the Atlantis Massif subseafloor (IODP Expedition 357): *Petrocella atlantisensis gen. nov.* sp. nov.

Marianne Quéméneur[1], Gaël Erauso[1], Eléonore Frouin[1], Emna Zeghal[1], Céline Vandecasteele[2], Bernard Ollivier[1], Christian Tamburini[1], Marc Garel[1], Bénédicte Ménez[3], Anne Postec[1]*

Supplementary Figures and Tables

A

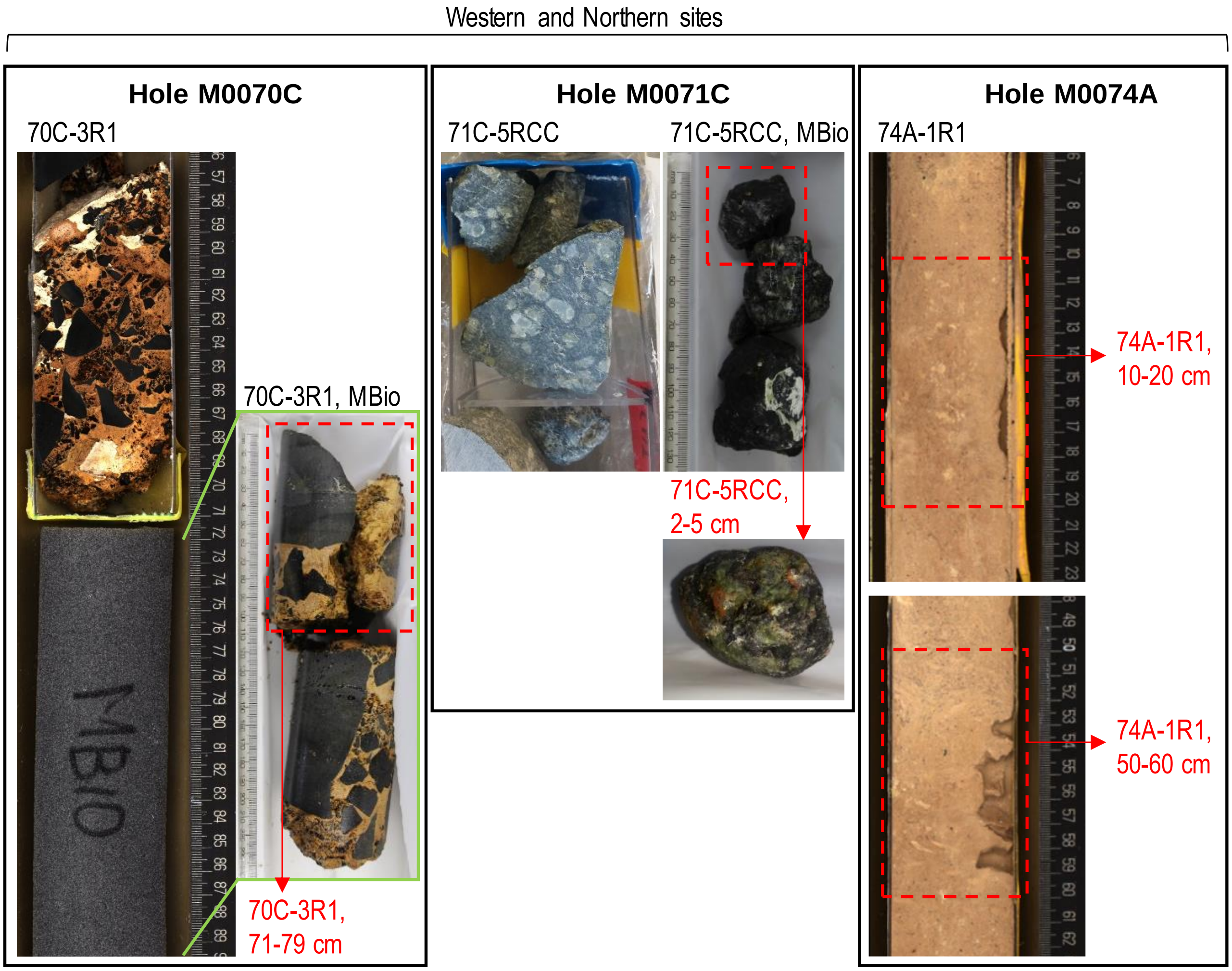

Supplementary Figure 1 (continued next page). Photographs of the IODP Expedition 357 core samples used in this study (Table 1). MBio refers to core sections sampled for microbiological characterization. (A) Western and Northern sites.

B

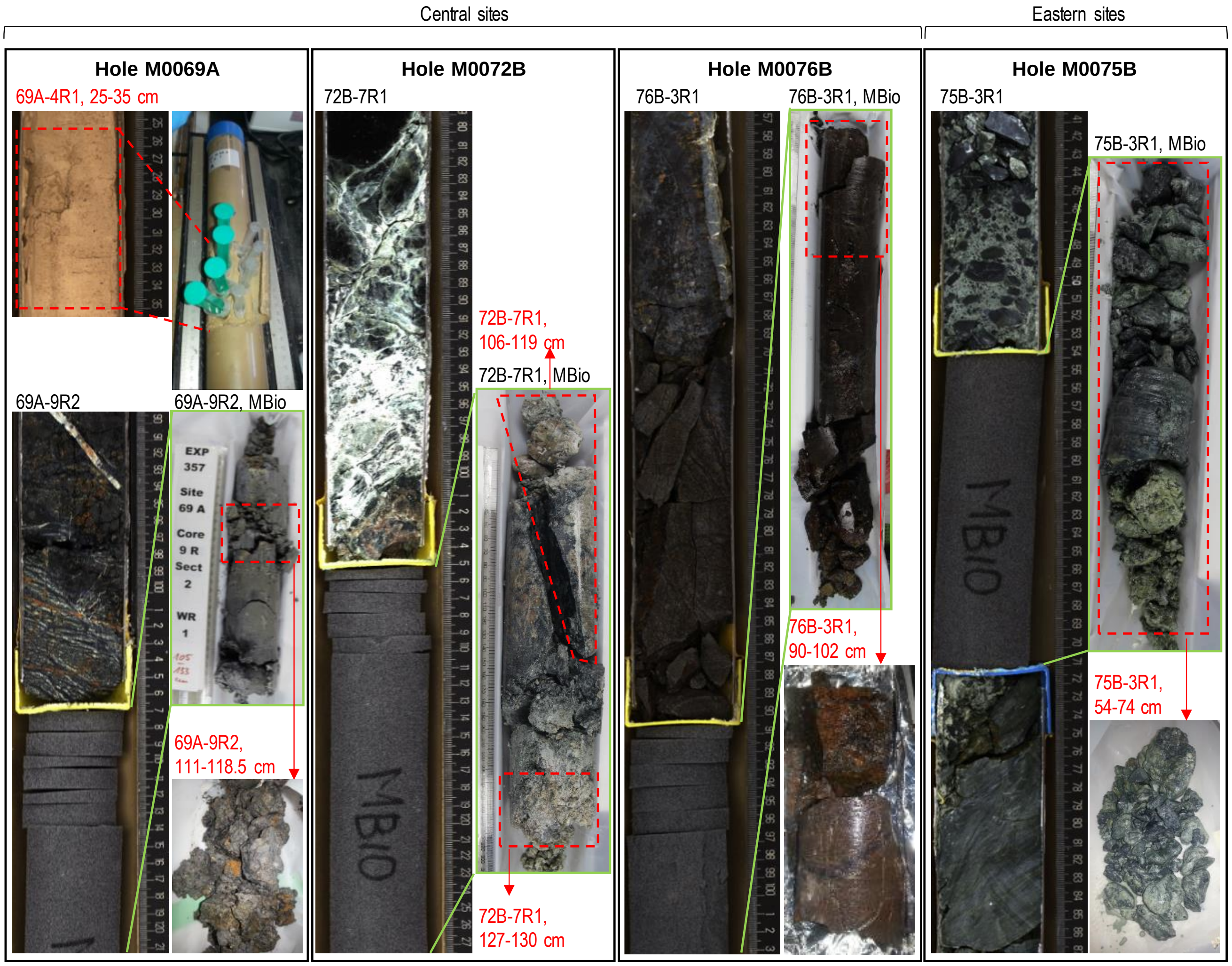

Supplementary Figure 1 (continued). Photographs of the IODP Expedition 357 core samples used in this study (Table 1). MBio refers to core sections sampled for microbiological characterization. (B) Central and Eastern sites.

A

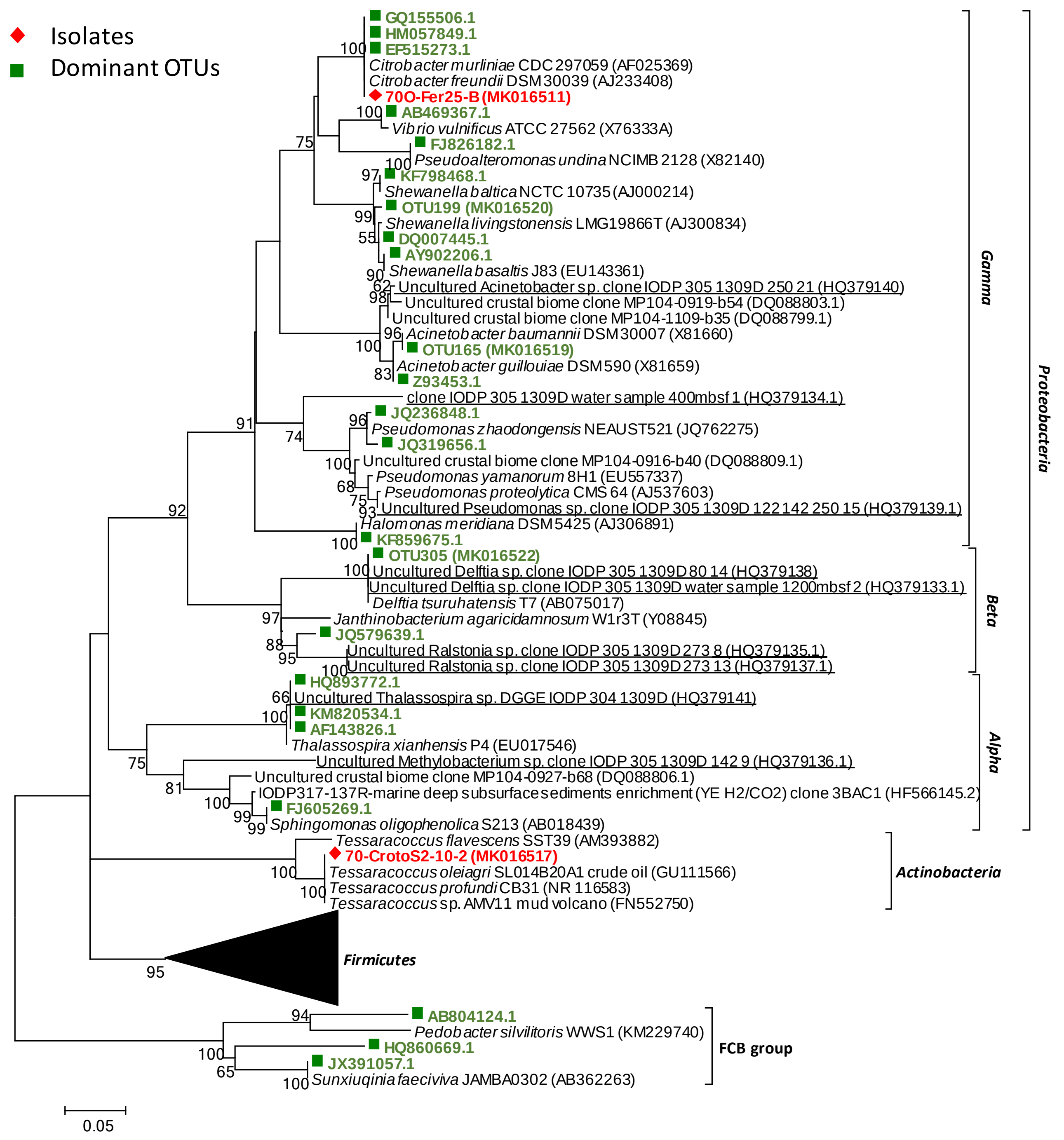

Supplementary Figure 2 (continued next page). Maximum likelihood phylogenetic trees based on 16S rRNA gene sequences showing the position of the bacterial isolates obtained in this study and the dominant OTUs (>10%) obtained in the enrichment cultures from rock samples cored in the Atlantis Massif. (A) All bacteria except *Firmicutes*, (B) *Firmicutes*. Clone sequences from IODP Expeditions 304 and 305 Hole 1309D (Atlantis Massif) are underlined (Mason et al., 2010). The Kimura 2 parameter was used, and the analysis involved 110 sequences and 386 aligned base pairs. Bootstrap values higher than 50% (based on 1,000 replicates) are shown at branch nodes. Accession numbers of type species are indicated in parentheses. Bar, in substitutions per nucleotide.

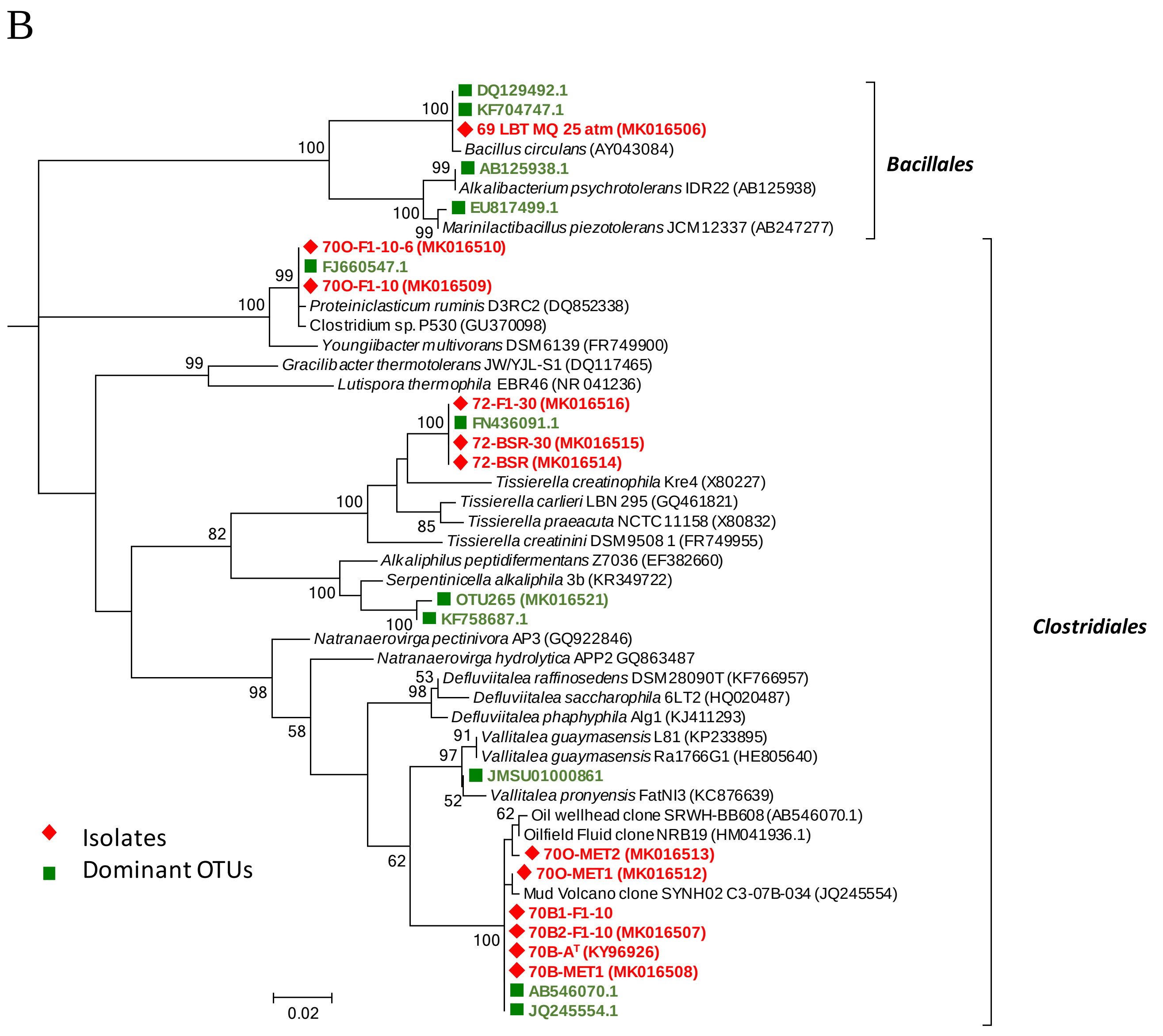

Supplementary Figure 2 (continued). Maximum likelihood phylogenetic trees based on 16S rRNA gene sequences showing the position of the bacterial isolates obtained in this study and the dominant OTUs (>10%) obtained in the enrichment cultures from rock samples cored in the Atlantis Massif. (A) All bacteria except *Firmicutes*, (B) *Firmicutes*. Clone sequences from IODP Expeditions 304 and 305 Hole 1309D (Atlantis Massif) are underlined (Mason et al., 2010). The Kimura 2 parameter was used, and the analysis involved 110 sequences and 386 aligned base pairs. Bootstrap values higher than 50% (based on 1,000 replicates) are shown at branch nodes. Accession numbers of type species are indicated in parentheses. Bar, in substitutions per nucleotide.

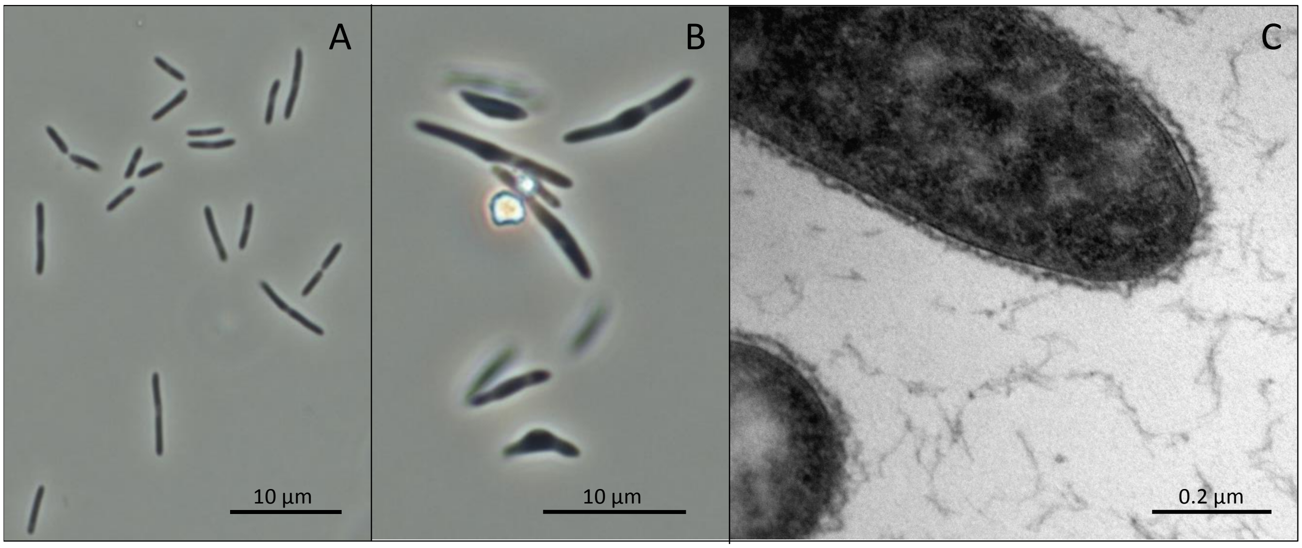

Supplementary Figure 3. Phase contrast microphotographs of strain 70B-A^T in exponential growth phase (A), under stress conditions with inflated and deformed cells (B), and ultrathin section transmission electron microscopy micrograph showing the absence of outer membrane typical of a Gram-positive cell wall (C).

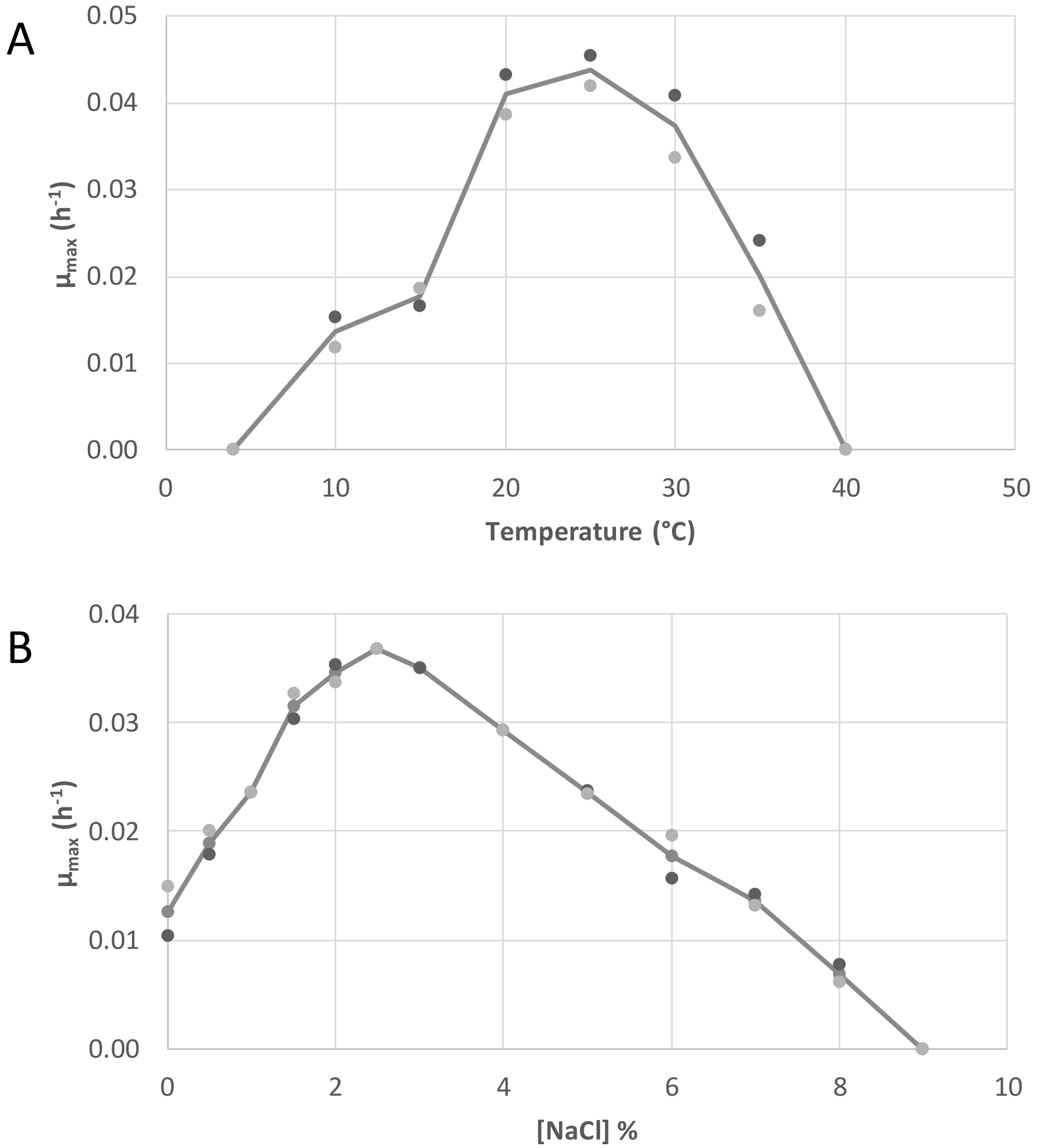

Supplementary Figure 4. Growth of strain 70B-A^T according to temperature (A) and NaCl concentration (B). The two maximum specific growth rate μ_{max} (h^{-1}) of duplicated cultures are shown with mean curves. Maximal growth was observed at 25°C (range 10 – 35°C) and 2.5% (w/v) of NaCl (range 1 – 8 %).

Supplementary Table 1. Culture media composition. F, SRB, MET and FE correspond to onshore culture media targeting fermenters, sulfate reducers, methanogens and iron-reducers, respectively. BM = Basal Medium used for strain 70B-A^T physiological characterization. F, MAcH2, MH2, MMet, SAc and SH2 correspond to onboard culture media targeting fermenters, acetotrophic and/or hydrogenotrophic and methylotrophic methanogens, and acetotrophic and hydrogenotrophic sulfate reducers.

	Onshore culture media								Onboard culture media					
**Component**	**F1**	**F2[1]**	**SRB1**	**SRB2[2]**	**MET1**	**MET2[2]**	**FE**	**BM**	**F**	**MAcH2[3]**	**MH2**	**MMet**	**SAc**	**SH2**
**Acetate (g l^{-1})**	1	0.1	1.6	-	1.6	-	1.6	-	-	1.6	-	-	1.6	-
**Balch oligo elements solution (ml l^{-1})[4]**	-	-	-	-	1	1	-	-	10	10	10	10	-	-
**$CaCl_2$ (g l^{-1})**	0.1	0.1	0.1	0.1	0.1	0.1	0.1	0.1	0.1	0.1	0.1	0.1	0.1	0.1
**Crotonate (g l^{-1})**	1	0.1	-	-	-	-	-	-	-	-	-	-	-	-
**Fe, Ni, Wo, Se solution (ml l^{-1})[5]**	1	1	1	1	1	1	1	-	-	1	1	1	-	-
**Formate (mM) or H_2CO_2 (80/20 v/v 2 bars)**	-	-	-	40	-	40	-	-	-	H_2CO_2[3]	H_2CO_2	-	-	H_2CO_2
**Iron(III) citrate (mM)**	-	-	-	-	-	-	5	-	-	-	-	-	-	-
**KCl (g l^{-1})**	0.3	0.3	0.3	0.3	0.3	0.3	0.3	0.3	0.1	0.1	0.1	0.1	0.1	0.1
**KH_2PO_4 (g l^{-1})**	0.3	0.3	0.3	0.3	0.3	0.3	0.3	0.3	0.3	0.3	0.3	0.3	0.3	0.3
**Lactate (mM)**	-	-	20	-	-	-	10	-	-	-	-	-	-	-
**L-Cysteine·HCl (g l^{-1})**	0.5	0.5	0.5	0.5	0.5	0.5	0.044	-	0.5	0.5	0.5	0.5	-	-
**Methanol (mM)**	-	-	-	-	30	-	-	-	-	-	-	30	-	-
**$MgCl_2·6H_20$ 50 g l^{-1} (ml l^{-1})**	20	20	20	20	20	20	20	20	2	2	2	2	2	2
**Na_2CO_3 8%**	Up to required pH													
**Na_2S 3% (ml l^{-1})**	20	20	20	20	20	20	-	20	20	20	20	20	20	20
**Na_2SO_4 (g l^{-1})**	-	-	3	3	-	-	0.1	-	-	-	-	-	3	3
**NaCl (g l^{-1})**	10	10	10	10	10	10	10	25	20	20	20	20	20	20
**NH_4Cl (g l^{-1})**	0.5	0.5	0.5	0.5	0.5	0.5	0.5	0.5	0.1	1	1	1	1	1
**Peptone (g l^{-1})**	1	0.1	-	-	-	-	-	-	-	-	-	-	-	-
**Pyruvate (mM)**	-	-	-	-	-	-	10	-	-	-	-	-	--	
**Resazurine (0.1 %) (ml l^{-1})**	0.3	0.3	0.3	0.3	0.3	0.3	-	0.3	0.3	0.3	0.3	0.3	0.3	0.3
**Trimethylamine (mM)**	-	-	-	-	10	-	-	-	-	-	-	-	-	-
**Tris (g l^{-1})**	2.4	2.4	2.4	2.4	2.4	2.4	2.4	2.4	3	3	3	3	3	3
**Tryptone (g l^{-1})**	1	0.1	0.1	0.1	0.1	0.1	0.1	-	2	-	-	-	0.5	0.5
**Widdel trace element solution (ml l^{-1})[6]**	1	1	1	1	-	-	1	-	-	-	-	-	1	1
**Yeast extract (g l^{-1})**	1	0.1	0.1	0.1	0.1	0.1	0.1	0.05	2	0.5	0.5	0.5	0.5	0.5

[1] F2 only used for enrichment culture at 0.1 MPa; [2] H_2CO_2 in cultures at 0.1 MPa, replaced by formate in HP cultures; [3] H_2CO_2 was added in cultures from M0069A-9R2; [4](Balch et al., 1979); [5]Fe, Ni, Wo, Se solution contained (l^{-1}): $FeSO_4·7\ H_2O$ 1.42 g, $NiSO_4·6H_2O$ 1.6 g, $Na_2WO_4·2\ H_2O$ 38 mg and $Na_2SeO_3·5\ H_2O$ 3 mg: [6](Widdel and Pfennig, 1981).

Supplementary Table 2. Sample name used in this study and corresponding description in Sequence Read Archive (SRA), with core sample data, culture media and conditions used for enrichment cultures (Description of the culture media can be found in Supplementary Table 1 and precise depths of the core samples are detailed in Supplementary Figure 1).

Name used in this study	Description in SRA	IODP Expedition 357 core sample	Culture medium	Growth temperature (°C)	Hydrostatic pressure for incubation (MPa)	Biological replicate / subculture
69A-MAcH2-25-0.1MPa-1	MQ1	69A-9R2	MAcH2	25	0.1	MQ replicate 1
69A-MAcH2-25-0.1MPa-2	MQ2	69A-9R2	MAcH2	25	0.1	MQ replicate 2
69A-MAcH2-25-0.1MPa-3	MQ3	69A-9R2	MAcH2	25	0.1	MQ replicate 3
69A-MAcH2-25-0.1MPa-4*	MQ4	69A-9R2	MAcH2	25	0.1	MQ replicate 4
69A-MAcH2-25-0.1MPa-5*	MQ5	69A-9R2	MAcH2	25	0.1	MQ replicate 5
69A-MAcH2-25-0.1MPa-6*	MQ6	69A-9R2	MAcH2	25	0.1	MQ replicate 6
69A-MAcH2-25-0.1MPa-7*	MQ7	69A-9R2	MAcH2	25	0.1	MQ replicate 7
69A-MAcH2-25-0.1MPa-8*	MQ8	69A-9R2	MAcH2	25	0.1	MQ replicate 8
69A-MAcH2-25-0.1MPa-9*	MQ9	69A-9R2	MAcH2	25	0.1	MQ replicate 9
69A-MAcH2-25-0.1MPa-10*	MQ10	69A-9R2	MAcH2	25	0.1	MQ replicate 10
69A-MAcH2-25-0.1MPa-11*	MQ11	69A-9R2	MAcH2	25	0.1	MQ replicate 11
69A-MAcH2-25-0.1MPa-12*	MQ12	69A-9R2	MAcH2	25	0.1	MQ replicate 12
69A-FE-10-0.1MPa	69.FE.1.ATM10	69A-9R2	FE	10	0.1	
69A-FE-25-0.1MPa	IODP.2	69A-9R2	FE	25	0.1	
71C-FE-25-0.1MPa	IODP.4	71C-5RCC	FE	25	0.1	
72B-FE-25-0.1MPa	IODP.5	72B-7R1	FE	25	0.1	
72B-H2CO2-30-0.1MPa	72.H2CO2.30degreesC	72B-7R1	MET2	30	0.1	
72B-F1-10-0.1MPa	72.F1.2.ATM10	72B-7R1	F1	10	0.1	
72B-F1-10-8.2MPa	72.2.F1.8.2MPa	72B-7R1	F1	10	8.2	
72B-SRB1-10-8.2MPa	72.2.BSR1.8.2MPa	72B-7R1	SRB1	10	8.2	
74A-FE-25-0.1MPa	IODP.6	74A-1R1	FE	25	0.1	
75B-F1-10-14MPa	75.1.F1.14MPa	75B-3R1	F1	10	14.0	
75B-SRB1-10-14MPa	75.1.BSR1.14MPa	75B-3R1	SRB1	10	14.0	
70C-F1-10-0.1MPa	70O.F1.ATM10	70C-3R1 carbonate subsample	F1	10	0.1	
70C-F2-25-0.1MPa	F2	70C-3R1	F2	25	0.1	F2
70C-F2-25-0.1MPa-rep	F2REP	70C-3R1	F2	25	0.1	F2 subculture 1
70C-FE-25-0.1MPa	FER1	70C-3R1	FE	25	0.1	FER1
70C-FE-25-0.1MPa-rep	FER1rep	70C-3R1	FE	25	0.1	FER1 subculture 1
70C-FE-25-0.1MPa-2	FER2	70C-3R1	FE	25	0.1	FER2
70C-FE-25-0.1MPa-2rep	FER2REP	70C-3R1	FE	25	0.1	FER2 subculture 1
70C-FE-10-0.1MPa	70O.FE.2.ATM10	70C-3R1 carbonate subsample	FE	10	0.1	
70C-FE-25-0.1MPa-3	IODP.3	70C-3R1	FE	25	0.1	replicate 1

70C-MET1-25-0.1MPa	MET1	70C-3R1	MET1	25	0.1	MET1
70C-MET1-25-0.1MPa-rep	MET1REP	70C-3R1	MET1	25	0.1	MET1 subculture 1
70C-MET2-25-0.1MPa	MET2	70C-3R1	MET2	25	0.1	MET2
70C-MET2-25-0.1MPa-rep	MET2REP	70C-3R1	MET2	25	0.1	MET2 subculture 1
70C-F1-10-14MPa-1	70B.P.F1.1	70C-3R1 basaltic subsample	F1	10	14.0	70B.P.F1. replicate 1
70C-F1-10-14MPa-2	70B.P.F1.2	70C-3R1 basaltic subsample	F1	10	14.0	70B.P.F1. replicate 2
70C-F1-10-14MPa-3	70O.P.F1.1	70C-3R1 carbonate subsample	F1	10	14.0	70O.P.F1.replicate 1
70C-F1-10-14MPa-4	70O.P.F1.2	70C-3R1 carbonate subsample	F1	10	14.0	70O.P.F1.replicate 2
70C-FE-10-14MPa-1	70O.P.FE.1	70C-3R1 carbonate subsample	FE	10	14.0	
70C-FE-10-14MPa-2	70B.P.FE.1	70C-3R1 basaltic subsample	FE	10	14.0	
70C-FE-10-14MPa-3	REPIQ.70B.Fe.14MPa	70C-3R1	FE	10	14.0	70B.Fe.14MPa subculture 1
70C-MET1-10-14MPa	70B.1.MET1.14 MPa	70C-3R1 basaltic subsample	MET1	10	14.0	70B.1.MET.14MPa replicate 1
70C-MET2-10-14MPa-1	70B.1.MET2.14 MPa	70C-3R1 basaltic subsample	MET2	10	14.0	70B.1.MET.14MPa replicate 2
70C-MET2-10-14MPa-2	70B.P.MET2.1	70C-3R1 basaltic subsample	MET2	10	14.0	70B.P.MET2. replicate 1
70C-MET2-10-14MPa-3	70B.P.MET2.2	70C-3R1 carbonate subsample	MET2	10	14.0	70B.P.MET2. replicate 2
70C-MET2-10-14MPa-4	70O.P.MET2.1	70C-3R1 carbonate subsample	MET2	10	14.0	70O.P.MET2. replicate 1
70C-MET2-10-14MPa-5	70O.P.MET2.2	70C-3R1 carbonate subsample	MET2	10	14.0	70O.P.MET2. replicate 2
70C-SRB1-10-14MPa	70B.1.BSR1.14MPa	70C-3R1 basaltic subsample	SRB1	10	14.0	
70C-SRB2-10-14MPa	70B.2.BSR2.14MPa	70C-3R1 basaltic subsample	SRB2	10	14.0	

***** 69A-MAcH2-25-0.1MPa-4 to 69A-MAcH2-25-0.1MPa-12 (corresponding to MQ4 to MQ12 in SRA) are not presented in this paper due the very high similarity of their microbial community composition with 69A-MAcH2-25-0.1MPa-3. mbsf stands for meters below seafloor. mbsl stands for meters below sea level.

Supplementary Table 3. Fatty acids compositions (given in percentage) of strain 70B-A^T and type strains of related genera: *V. guaymasensis* ^{T} (Lakhal et al., 2013) and *N. pectinivora* ^{T} (Sorokin et al., 2012). The dominant fatty acids (> 10% of the total fatty acids) are indicated in bold for each strain.

**Fatty acids**	**70B-A^T**	***V. guaymasensis*** ^{T}	***N. pectinivora*** ^{T}
**iso DMA-C15:0**	**-**	**10.0**	**-**
**anteiso C15:0**	0.28	**22.7**	**-**
**anteiso DMA-C15:0**	**-**	**10.8**	**-**
**iso C15:0**	0.19	**13.7**	1.6
**DMA C16:0**	2.45	5.5	-
**C16:0**	**22.1**	7.0	**46.9**
**C16:1 w7c**	**35.6**	-	**11.5**
**C16:1 w7c DMA***	**14.3**	-	-
**C18:1 w7c**	8.2	-	**17.5**
**C18:1 w7t**	-	-	6.0

*DMA = dimethyl acetals

Supplementary References

Balch, W. E., Fox, G. E., Magrum, L. J., Woese, C. R., and Wolfe, R. S. (1979). Methanogens: reevaluation of a unique biological group. *Microbiol. Rev.* 43**,** 260-296.

Lakhal, R., Pradel, N., Postec, A., Hamdi, M., Ollivier, B., Godfroy, A., et al. (2013). *Vallitalea guaymasensis* gen. nov., sp. nov., isolated from marine sediment. *Int. J. Syst. Evol. Microbiol.* 63**,** 3019-3023. doi: 10.1099/ijs.0.045708-0.

Mason, O. U., Nakagawa, T., Rosner, M., Van Nostrand, J. D., Zhou, J., Maruyama, A., et al. (2010). First investigation of the microbiology of the deepest layer of ocean crust. *PLoS ONE* 5(11)**,** e15399. doi: 10.1371/journal.pone.0015399.

Sorokin, D., Tourova, T., Panteleeva, A., Kaparullina, E., and Muyzer, G. (2012). Anaerobic utilization of pectinous substrates at extremely haloalkaline conditions by *Natranaerovirga pectinivora* gen. nov., sp. nov., and *Natranaerovirga hydrolytica* sp. nov., isolated from hypersaline soda lakes. *Extremophiles* 16(2)**,** 307-315. doi: 10.1007/s00792-012-0431-6.

Widdel, F., and Pfennig, N. (1981). Studies on dissimilatory sulfate-reducing bacteria that decompose fatty acids. I. Isolation of new sulfate reducing bacteria enriched with acetate from saline environments. Description of *Desulfobacter postgatei* gen. nov., sp. nov. *Arch. Microbiol.* 129**,** 395-400. doi: 10.1007/BF00406470.
